# Assessing 16 Fundamental Motives With Fewer Than 50 Items: Development and Validation of the German 16 Motives Research Scales (16mrs)

**DOI:** 10.3389/fpsyg.2021.562371

**Published:** 2021-03-08

**Authors:** Jan Dörendahl, Samuel Greiff, Christoph Niepel

**Affiliations:** Cognitive Science & Assessment, University of Luxembourg, Esch-sur-Alzette, Luxembourg

**Keywords:** fundamental motives, short scales, personality assessment, motivation, scale development

## Abstract

Psychometrically sound short scales are required to comprehensively and yet economically assess fundamental motives in research settings such as large-scale assessments. In order to provide such a time- and cost-efficient instrument, we conducted three studies (*N* = 1,568) to develop further and validate 16 German scales with three items each assessing fundamental motives [16 motives research scales (16mrs)]. In Study 1, we applied a top–down construction process to develop a preliminary item pool on the basis of a thorough revision of existing construct definitions. In Study 2, we chose an approach that allowed us to balance the optimization of psychometric properties with content coverage to select three-item scales for each of the 16 motives. For the item selection process, we combined exploratory factor analyses, confirmatory factor analyses, ant colony optimization algorithm, and Mokken scale analyses. In Study 3, we cross-validated the results obtained in Study 2 and placed the 16mrs in a nomological network consisting of Big Five traits and Power, Achievement, Affiliation, Intimacy, and Fear motives. The results of these studies indicate that the 16mrs can be used to reliably and validly assess fundamental motives that represent a level of personality that differs from the Big Five and covers motivational aspects beyond the well-established Power, Achievement, Affiliation, Intimacy, and Fear motives. Limitations concerning the reliability of the Autonomy scale and the empirical discrimination of the Dominance and Status scales are discussed. In addition to the validated German version, we also provide the English translation of the items, which, however, need to be validated before use.

## Introduction

In motive research, a distinction is typically made between two independent motive systems: implicit motives and explicit motives (McClelland et al., 1989). Implicit motives are usually conceived as unconscious affective preferences for certain types of incentives that cannot be verbalized and therefore require the use of projective assessment techniques, such as the thematic apperception test (McClelland et al., 1989; Schönbrodt and Gerstenberg, 2012). Explicit motives can be measured with questionnaires and are defined as people’s self-concepts about their goals, values, and affective preferences (Schönbrodt and Gerstenberg, 2012). While implicit motives predict behavior in open-ended situations, explicit motives predict behavior in structured situations, such as making decisions or forming evaluations (Brandstätter et al., 2013). Although various taxonomies of explicit motives exist (e.g., Murray, 1938), scholars have yet to agree on a theoretically meaningful, self-contained motive classification that covers motivational aspects beyond the Big Three (i.e., Power, Achievement, and Affiliation; Heckhausen and Heckhausen, 2010). The overemphasis of the Big Three comes at the expense of neglecting motivational constructs that are not covered by the Big Three, for instance, curiosity or order, thus arguably impeding a comprehensive understanding of human motivation (Bilsky, 2006). To propose a more fine-grained, theoretically meaningful, self-contained framework of motives, fundamental motives were introduced as a specific subset of explicit motives that are not pursued for instrumental purposes but rather for what people ultimately want (Reiss and Havercamp, 1998). According to Reiss and Havercamp (1998), explicit motives not included in the subset of fundamental motives may very well be pursued for instrumental purposes. For instance, if people strive for a well-paying job because it grants them recognition and status in society, the act of striving for a well-paying job can be considered instrumental because it is a means to attain something beyond itself (Reiss, 2004). In general, motives cover motivational aspects of human personality that are not described by traits such as the Big Five (i.e., Extraversion, Agreeableness, Conscientiousness, Negative Emotionality, and Openmindedness) personality factors because the latter are seen as encompassing observed patterns of human behavior, but they do not capture the reasons for the behavior (Bilsky and Schwartz, 1994; McAdams and Olson, 2010). Thus, people who score high on extraversion, as defined in the Big Five Inventory-2 (BFI-2; Soto and John, 2017), are described as exhibiting sociable, assertive, and enthusiastic behavior, which is likely to have value in predicting future behavior. However, this description does not explain why people behave in such a way. Nevertheless, covering aspects of why people behave the way they do is indeed important, for instance, to obtain insights into the setting and attaining of goals, which are described by Freund and Riediger (2006) as major processes in life. As such, they involve a flexible *personality-in-context* that interacts with the environment over time. The aspect of goal attainment is arguably better represented by psychological constructs such as motives because they take current situational and environmental aspects into account, for instance, the cues that activate a behavior that targets the satisfaction of a motive (Freund and Riediger, 2006). Traits, on the other hand, explicitly do not cover contextual aspects that can change because they focus on descriptions of *trans*-situationally stable patterns of behavior (Bilsky, 2006; McAdams and Olson, 2010).

Reiss and Havercamp (1996) applied a two-step approach to theoretically identify fundamental motives out of the basically infinite number of explicit motives. First, they compiled a list of explicit motives based on theoretical considerations. They reviewed the motive literature, existing frameworks such as the system of needs (Murray, 1938), and existing questionnaires such as the personality research form (Jackson, 1974). Subsequently, they extracted all explicit motives exhibiting four properties. First, the motives should be fundamental motivators, that is, motives are considered fundamental if they cannot be explained by or reduced to other motives. Second, motives should allow predictions to be made about people’s behaviors and habits on the basis of the strength of the respective motives (Reiss and Havercamp, 1996). Third, motives should allow individual differences to exist, that is, the meaning of particular classes of incentives may vary across individuals. Fourth, motives should account for a considerable amount of everyday behavior (Reiss, 1999), while no motives that exclusively reflect bodily needs (e.g., thirst) are included. In a second step, the theoretically derived list of 25 potential fundamental motives (Reiss and Havercamp, 1996) was empirically tested. To this end, based on this list, items were developed to assess each of these motives (Havercamp, 1998; Reiss and Havercamp, 1998). After four exploratory factor analyses and one confirmatory factor analysis were conducted, 16 factors emerged (Havercamp, 1998; Reiss and Havercamp, 1998). The factors were labeled according to the contents of the items that loaded on them (Table 1). In sum, fundamental motives represent an intriguing approach to assessing explicit motives, as the framework combines comprehensiveness in terms of the included motives with comprehensibility in the form of the four rules about which motives to include. Hence, the fundamental motives represent a theoretically meaningful, self-contained framework that allows for the investigation of largely neglected motives, such as idealism or structure, beyond the so-called “Big Three” of motivation (i.e., power, achievement, and affiliation; Bilsky, 2006; Heckhausen and Heckhausen, 2010).

**TABLE 1:** The 16 fundamental motives and their respective construct definitions.

**Fundamental motive**	**Original definition**	**Revised definition**
Curiosity	Need to gain knowledge	Interest in increasing one’s knowledge, gaining perceptions, and seeking intellectual challenges
Social Acceptance (Acceptance)	Need for the approval of others	Being concerned with getting recognition from other people and being accepted by them
Dominance (Power)	Need to influence and lead	Being concerned with having an impact on other people and influencing people as well as processes
Status	Need for high social status	Being concerned with getting and maintaining a reputation and a prominent position in society
Retention (Saving)	Need for frugality and collecting	Being concerned with building up stocks and maintaining them
Autonomy (Independence)	Need for autonomy	Being concerned with being independent from other people’s impact and expectations
Social Participation (Social Contact)	Need for the company of other people	Seeking the company of and being interested in other people
Morality (Honor)	Need to follow traditional rules	Being concerned with social norms that apply to society and the need to comply with them
Idealism	Need for justice and altruism	Being concerned with helping disadvantaged people and improving society
Structure (Order)	Need for organizing and rituality	Being concerned with organizing and structuring one’s environment in a simple and consistent manner
Safety (Tranquility)	Need to avert anxiety and fear	Being concerned with having a peaceful and secure life
Revenge (Vengeance)	Need for revenge	Being concerned with retaliating when wronged or insulted by others
Physical Exercise	Need to exercise the body	Being concerned with physical activity and exercise
Food Enjoyment (Eating)	Need for food	Being concerned with having pleasurable experiences while eating food. This motive goes beyond the bodily need of eating
Family	Need for raising offspring	Being concerned with providing care for one’s family. The motive mainly refers to one’s family of origin but might also include one’s partner or offspring
Sex (Romance)	Need for courting and having sex	Being concerned with having sensual and erotic experiences as well as an active, fulfilling sex life

*The original definitions were adapted from Reiss (2004) after careful consideration of the existing theoretical and empirical literature on motives. In the course of the revision of the construct definitions, we also revised some of the motive labels to better reflect the revised definition. Terms in parentheses refer to those labels used by Reiss (2004) that differ from our labels.*

Investigations of convergent and divergent correlations between fundamental motives and other personality frameworks have yielded results that have helped clarify the distinction between motives and traits. For instance, Olson and Weber (2004) investigated the relationships between the 16 fundamental motives and the traits in the five-factor model (FFM) of personality (Costa Jr., and McCrae, 1992). The results showed that correlations between fundamental motives and Big Five traits were usually small to medium in size, with only three out of 80 correlations exceeding the threshold for a large effect, that is, *r* > 0.50 (Cohen, 1988). Thus, on the basis of the overall results, the authors argued that fundamental motives and traits have only a moderate overlap, thus providing support for the argument of McAdams (1995) and Winter et al. (1998) that motives and traits are both needed to achieve a comprehensive conceptualization of personality. Thus, in sum, motives provide incremental validity when predicting behavior or life outcomes and better describe the interaction between personality characteristics on the one hand and situational and environmental characteristics that are necessary for the attainment of goals on the other (*personality-in-context*; Bilsky, 2006; Freund and Riediger, 2006).

To utilize fundamental motives in an investigation, a questionnaire that can be used to assess the large number of 16 separate constructs is needed. If it additionally does so in an economic fashion, it can be used when time and monetary resources are (severely) limited, such as in large-scale assessments (Rammstedt and Beierlein, 2014). Therefore, the call for properly constructed psychological short scales has recently become louder (Rammstedt and Beierlein, 2014), and many researchers would greatly appreciate a short but still psychometrically sound tool for assessing the 16 fundamental motives. Although the topic of short scales is somewhat controversial (Ziegler et al., 2014), there are research settings where short scales are preferable, for instance, in large-scale assessments, as already mentioned (Rammstedt and Beierlein, 2014), but also online assessments in general. Previous research has indicated that when conducting lengthy assessments, there is always the risk of participant dropout or decrease in the response rate, especially when carried out *via* the Internet (Edwards et al., 2004; Hoerger, 2010; Sandy et al., 2014). As a consequence, researchers usually try to avoid questionnaires that were constructed for individual assessment and decision-making because such questionnaires tend to have very good reliabilities but come at the cost of more items that take longer to answer (Rammstedt and Beierlein, 2014). However, for the assessment of the 16 fundamental motives, the only existing assessment tools are primarily used for individual assessment and decision-making. Specifically, these are a royalty-based questionnaire called the Reiss Profile (Havercamp, 1998; Reiss and Havercamp, 1998) and a questionnaire that was recently developed for counseling purposes called the LUXXprofile (Kemper et al., 2017). The Reiss Profile’s 16 scales consist of eight items each and show test–retest correlations ranging from 0.69 to 0.88 with a mean of 0.80 for a 4-week interval (Havercamp, 1998). The LUXXprofile scales, with nine items each, demonstrate internal consistencies ranging from 0.62 to 0.90 with a mean of 0.84 (Kemper et al., 2017). Both measures have been validated and take approximately 15 min to complete (Havercamp, 1998; Kemper et al., 2017). Now, the considerable methodological advances (Olaru et al., 2015) that have been made in the field of short scale construction in the 20 years that have passed since the publication of the Reiss Profile have provided the armamentarium needed to develop the much appreciated short scales for the assessment of fundamental motives in research settings, which is the aim of the current study.

We conducted three studies to newly develop, construct, and validate the 16 motives research scales (16mrs), a brief questionnaire for the assessment of 16 fundamental motives mainly based, among others, on the framework by Havercamp (1998) and Reiss (2004, 2008). Our first aim was to find a balance between shortness and desirable psychometric properties. That is, we wanted the scales to have reliabilities that would be sufficient for the assessment of group differences, rendering the short scales suitable for research purposes. The second aim was to validate the 16mrs and investigate its nomological network. To this end, we analyzed the relationships between 16 fundamental motives as assessed by the 16mrs and the Big Five personality traits in addition to the explicit Power, Affiliation, Achievement, Intimacy, and Fear motives, which have been extensively investigated in motivational research (Heckhausen and Heckhausen, 2010). To address the first aim, we conducted the preliminary Studies 1 and 2. In Study 1, we developed an item pool and selected preliminary scales. In Study 2, we revised the item pool on the basis of the results of Study 1 by modifying or removing problematic items and adding newly developed items before we selected the final scales for the 16mrs. To address the second aim, we conducted Study 3. Here, we cross-validated the results from Study 2 and computed convergent and discriminant validities for the 16mrs with the Big Five personality traits and the explicit Power, Affiliation, Achievement, Intimacy, and Fear motives. To make the fully validated 16mrs available, we present its 16 scales (encompassing three positively keyed items each) in the Supplementary Material.

## Studies 1 and 2

**FIGURE 1:**
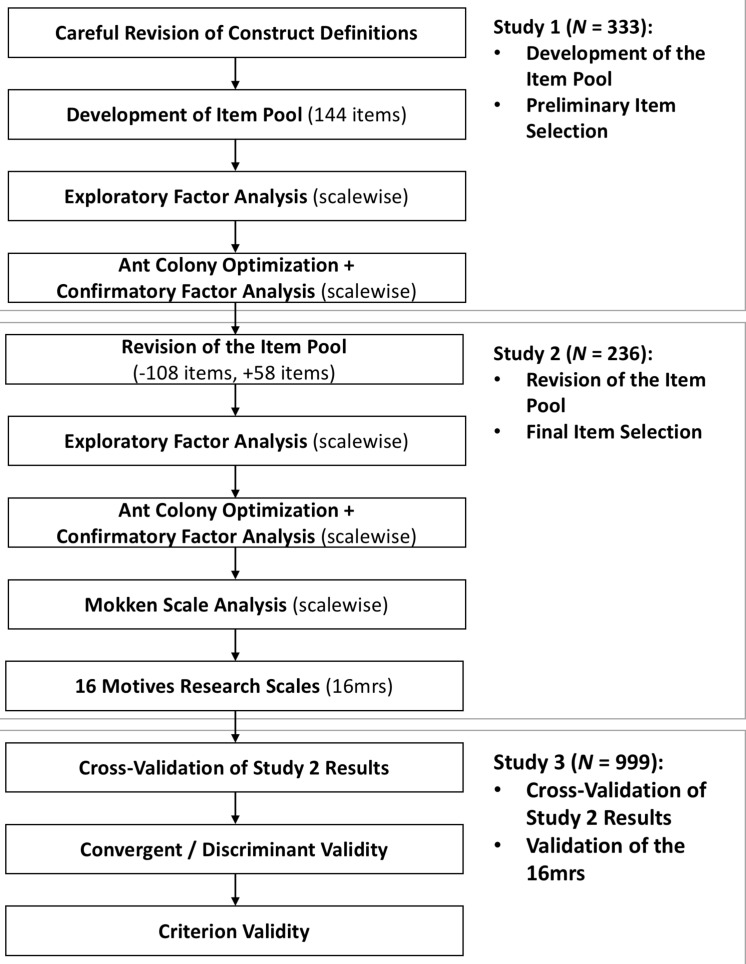
Overview of the construction and validation process of the 16 motives research scales (16mrs). Additional detailed information about Studies 1 and 2 is provided in the Supplementary Information. Study 3 is described below.

Over the course of two preliminary studies, we constructed 16 short scales with three positively keyed items each for the assessment of fundamental motives in research settings (Figure 1). We decided to use only positively keyed items so that it would not be necessary to invert any items for the analyses. Further, a large body of research has demonstrated that including negatively keyed items in a scale introduces method variance (e.g., Lai, 1994; Ibrahim, 2001), which can decrease reliability (Lai, 1994). The construction process was based on two samples (*N* = 569) that can be considered representative of the German population with respect to age, gender, and education. In addition to the economic advantages, a short scale developed from scratch would additionally offer the opportunity to further improve the fit of items and construct definitions for fundamental motives on the basis of revised construct definitions as carried out in our research. Of note, there seems to be a mismatch between the original definitions of the 16 motives and the respective items (as far as they are published, for instance, in Havercamp, 1998 and as far as this can be evaluated given that not all items are published). For example, the definition of the Tranquility motive in its original version (Table 1) covers the avoidance of anxiety and fear (Reiss, 2004), whereas the items from the Reiss Profile also tap into the avoidance of pain and unpleasant body states (Havercamp, 1998). For the Power motive, two considerably different definitions exist. One definition (Reiss, 2004) reflects the Reiss Profile item contents well (Havercamp, 1998) because it covers the need to influence others and to lead (Table 1). However, in a different publication (Reiss, 2000), aspects such as seeking challenges and excellence, which seem to belong instead to the Achievement motive, were mixed into the definition of Power. Consequently, our aim in Study 1 was to carefully revise the existing construct definitions (Table 1) based on an extensive literature review (a complete list of the literature consulted for the construct definitions can be found in the Supplementary Material). On the basis of the revised construct definitions, we developed a pool of 144 items and conducted preliminary scale constructions using exploratory factor analysis and ant colony optimization (ACO) algorithm (Marcoulides and Drezner, 2003) in combination with confirmatory factor analysis. Based on the results, we revised the item pool. To this end, we removed 108 items and added 58 newly developed items.

The aim of Study 2 was to select the final 16mrs from the revised item pool. To this end, we repeated the procedure of conducting exploratory factor analysis and ACO in combination with confirmatory factor analysis to select the best four items for each scale. In a final step, we applied Mokken scale analysis (Mokken, 1971) so that, out of each set of four items, we could select the three that could best discriminate between people with a high and a low standing on the respective motive for the final scales. As the resulting scales satisfied the assumptions of the Mokken scale analysis (i.e., unidimensionality, local independence, and latent monotonicity), the models could be concluded to provide justification for using the scales’ sum scores to represent a person’s standing on the measured motive (Sijtsma and Molenaar, 2002). To provide precise estimations of each scale’s reliability, we computed the latent class reliability coefficient (LCRC; van der Ark et al., 2011). This approach is based on the joint density of an unconstrained latent class model (van der Ark, 2011, Van der Ark, 2012). In addition, we calculated the coefficient alpha. Although LCRC has been reported to produce more precise estimates of reliability (van der Ark et al., 2011), we also report the coefficient alpha to be able to compare the reliabilities reported in this study with those reported in other studies. Reliabilities of the final scales in terms of LCRC and coefficient alpha ranged from LCRC = 0.63 and α = 0.64 (Sex) to LCRC = 0.90 and α = 0.87 (Family), while the scalability coefficients *H* ranged from.42 (Sex) to.74 (Physical Exercise). Following Mokken’s (1971) rules of thumb, the constructed scales can be considered moderate to strong. Further information about the construction process and the results can be found in the Supplementary Material.

## Study 3

Our aim in Study 3 was to cross-validate the results that we obtained in Study 2 and to extensively validate the 16mrs. We considered a cross-validation reasonable, as ACO results have been found to provide an overfit in some cases (Olderbak et al., 2015) and we relied on ACO to a large extent throughout our construction procedure. To this end, we again examined scalability coefficients under the monotone homogeneity model (MHM; Mokken, 1971) using a large online sample that was representative of the German online population with respect to age and gender. Thus, we aimed to provide evidence that the scalability coefficients obtained in Study 2 could not be reduced to sample fluctuations and that the scores of the selected scales would maintain their psychometric properties in a different sample (Fokkema and Greiff, 2017). As the LUXXprofile (Kemper et al., 2017) scales were included as optimization criteria in Studies 1 and 2 (please see the Supplementary Material for further details), we compared the 16mrs scales with the corresponding LUXXprofile scales in a correlational analysis. Furthermore, to provide support for the scale’s criterion validity and investigate fundamental motives’ nomological network, participants completed a series of behavioral indicators (Table 2) as well as the Unified Motive Scales 6 (UMS-6; Schönbrodt and Gerstenberg, 2012) to assess explicit power, achievement, affiliation, intimacy, and fear motives. We developed hypotheses about the relationships between fundamental motives and UMS-6 motives we expected to be largest based on conceptual overlap. The remaining correlations were investigated in an exploratory fashion and are reported for the sake of completeness. The power motive is defined as concern about having influence and prestige (Heckhausen and Heckhausen, 2010). Hence, we expected the highest correlations to occur with Dominance and Status. For the achievement motive (i.e., the concern about mastering challenging tasks; Heckhausen and Heckhausen, 2010), we expected the highest correlations with the Curiosity, Dominance, and Status motives. The Affiliation motive is defined as the need for social contacts (Heckhausen and Heckhausen, 2010). Consequently, we expected the highest correlation to occur with the Social Participation motive. For the Intimacy motive (i.e., concern about having positive relationships with people close to oneself; Heckhausen and Heckhausen, 2010), we expected the highest correlation with the Family motive. The Fear motive is a general tendency to be afraid of failing to satisfy one’s motives (Schönbrodt and Gerstenberg, 2012). Here, we expected the highest correlation to occur with Social Acceptance.

**TABLE 2:** Behavioral indicators used to investigate the criterion validity of the 16 motives research scales (16mrs).

**Scale**	**Criterion**
Curiosity	Frequency of consulting the website Wikipedia per week as a source of information. We expected a positive relationship between Curiosity and the frequency of Wikipedia visits.
Dominance	Occupation of a leadership position in the job (none vs. at least one employee under their supervision). We hypothesized that Dominance would motivate people to try to obtain a work position that would allow them to supervise employees.
Status	Number of academic degrees (including Bachelor, Diploma, Master, and Doctorate). Because academic degrees have a certain prestige, we expected a positive relationship between Status and the number of academic degrees.
Retention	Sum of the financial investments that the participants reported using (e.g., savings account, life insurance, stocks). We expected that Retention scores would positively predict the number of financial investments.
Social Participation	Number of friends. We expected that Social Participation would positively predict the number of friends reported by the participants.
Morality	Delinquent behavior as assessed with three items used in ALLBUS (Gesis – Leibniz-Institut für Sozialwissenschaften, 2012). The three items asked people about the frequency of engaging in (a) fare evasion, (b) drunk driving, and (c) providing untrue information on their income tax statement. We computed the mean score across the items to assess the overall tendency to engage in delinquent behavior. We expected that Morality scores would negatively predict the tendency to engage in delinquent behavior.
Idealism	Having held an honorary office: yes/no. We expected that people with higher scores on Idealism would be more likely to have held an honorary office.
Safety	Working freelance: yes/no. We expected that participants with higher Safety scores were less likely to freelance because freelancing is associated with more risk in comparison with permanent employment.
Physical Exercise	Frequency of playing sports per week. We expected that participants with a higher score on Physical Exercise would report playing sports more often.
Food Enjoyment	Frequency of eating at a restaurant per month. We expected a positive relationship between Food Enjoyment and the frequency of restaurant visits.
Family	Frequency of visiting family members who do not live in the participant’s home. We expected that participants with higher scores on Family would report visiting family members more often.

We also administered the BFI-2 (Danner et al., 2016), which is used to assess the Big Five personality traits. Demonstrating empirically that the 16mrs assess different constructs of personality rather than traits is important because traits and motives are described as referring to different levels of personality (McAdams, 1995; Winter, 2005). This distinction should become evident in a pattern of results consisting mainly of small correlation coefficients with a few moderate correlation coefficients. Again, we derived hypotheses about the largest expected relationships and investigated the remaining correlations in an exploratory fashion and reported these results for the sake of completeness. Extraversion, as assessed with the BFI-2, is defined as being sociable, assertive, and full of energy. Here, we expected that the strongest positive correlation coefficients would occur with Social Participation, Dominance, and Physical Exercise motives. The BFI-2 facets of Agreeableness are Compassion, Respectfulness, and Trust. We expected the strongest correlations to occur for Idealism and Social Participation, as well as Revenge (negative correlation) and Social Acceptance (negative correlation). Conscientiousness encompasses the facets Organization, Productiveness, and Responsibility. Here, we expected the strongest correlations to occur with the Structure motive. Negative Emotionality is composed of the facets Anxiety, Depression, and Emotional Volatility. Hence, we expected the strongest correlations to occur with Social Acceptance. Open-mindedness is defined in the BFI-2 as comprising the facets Intellectual Curiosity, Aesthetic Sensitivity, and Creative Imagination. Consequently, we hypothesized the highest correlations to occur with the Curiosity motive.

### Method

#### Participants and Procedure

Participants were 999 adolescents and adults (48.7% young women and women) between the ages of 16 and 69 years (*M* = 43.13, *SD* = 14.88); 18 participants were not native German speakers; 15 of these participants reported having very good German language skills, and two reported having good German language skills. Only one reported having only moderate German language skills. This sample was used for the Mokken scale analysis. For the validity analysis, three non-overlapping subsamples were drawn from the complete sample, whereby the first subsample completed the UMS-6, the second completed the BFI-2, and the third completed the behavioral indicators.

Participants were sampled by the private survey institute forsa main^1^ based in Frankfurt/Main, Germany. This independent institute conducts surveys for research, political, and state institutions as well as for companies. Forsa is a member of ESOMAR^2^, which ensures that data collection and processing are conducted in an ethical manner. The data were fully anonymized by forsa before all authors had access to them. The participants were informed that the results of this survey would potentially be published. The sample and its three subsamples provided by forsa were representative of the German online population with respect to age and gender. Further sociodemographic information about the full sample and the subsamples is presented in the Supplementary Material. For the sampling procedure, forsa stratified its panel according to age and gender and then randomly selected participants until predefined quotas were met. After the participants completed the 16mrs and the sociodemographic items, in accordance with the predefined quotas, they were again assigned to one of three subsamples that completed either the BFI-2 or the UMS-6 for the investigation of convergent and discriminant validity or answered the behavioral indicators for criterion validity. To estimate convergent and discriminant validity, we used samples of *n*_1_ = 200 (*M*_*age*_ = 43.20, *SD* = 14.84, Range = 16–69) and *n*_2_ = 199 (*M*_*age*_ = 43.25, *SD* = 14.90, Range = 17–69). The calculation of criterion validity was based on *n*_3_ = 200 (*M*_*age*_ = 42.88, *SD* = 14.99, Range = 16–69). For job-related outcomes (i.e., the criteria for Dominance and Safety), we filtered the full data set to retrieve all people who were employed; this resulted in *n_working_* = 703 (*M*_*age*_ = 44.13, *SD* = 12.34, Range = 16–69).

Participants completed the survey online. First, they completed demographic questions asking for age, gender, and education. Afterward, they rated all 48 of the 16mrs items on a 6-point Likert scale ranging from 0 (*does not apply at all*) to 5 (*applies completely*). Subsequently, the participants were then assigned to one of the subsamples to complete the BFI-2, the UMS-6, or a series of items asking for everyday behavior. For the behavioral indicators, different item formats were used, for example, dichotomous item formats (e.g., “Have you ever taken a cooking course?”) with the options “yes” or “no” or open response formats (e.g., “How many hours per week do you play sports?”).

#### Measures

##### Big Five Inventory-2

We assessed the FFM traits with the German version (Danner et al., 2016) of the BFI-2 (Soto and John, 2017), which was completed by *n*_2_. Extraversion, Agreeableness, Conscientiousness, Negative Emotionality, and Open-mindedness were assessed with 12 items each. Participants rated each item on a 5-point Likert scale ranging from 1 (*disagree strongly*) to 5 (*agree strongly*). We scored each scale by averaging the items after the negatively keyed items had been reversed. In this sample, the scales’ alpha reliabilities were 0.86 (Extraversion), 0.81 (Agreeableness), 0.87 (Conscientiousness), 0.85 (Negative emotionality), and 0.86 (Open-mindedness).

##### Unified Motive Scales 6

We used the UMS-6 (Schönbrodt and Gerstenberg, 2012) to assess five motivational dimensions with six items each. The questionnaire was completed by *n_1_.* The explicit motives assessed by the UMS-6 are Power, Achievement, and Affiliation, which are commonly referred to as the Big Three (Heckhausen and Heckhausen, 2010), plus Intimacy and Fear. For the UMS-6, two different response options for the 6-point Likert scales were used as intended by Schönbrodt and Gerstenberg (2012). The 13 UMS-6 items that target goals required importance ratings and were rated on a Likert scale ranging from 0 (*not important to me*) to 5 (*extremely important to me*), whereas the 17 statements that required agreement ratings ranged from 1 (*strongly disagree*) to 6 (*strongly agree*). We scored each scale by averaging the items after the negatively keyed items had been reversed, as described by Schönbrodt and Gerstenberg (2012). Alpha reliabilities for the five scales in this sample were 0.91 (Power), 0.88 (Achievement), 0.89 (Affiliation), 0.80 (Intimacy), and 0.85 (Fear).

##### LUXXprofile

We assessed the 16 fundamental motives with the LUXXprofile (Kemper et al., 2017), consisting of 144 items. We are not able to provide further psychometric information for intellectual property reasons.

##### Behavioral indicators

To assess the criterion validity of the 16mrs, we had participants complete a variety of behavioral indicators. To this end, we collected specific behaviors associated with the respective fundamental motives that could be assessed *via* self-reports in an online survey. For some fundamental motives, finding suitable criteria was straightforward (e.g., hours per week spent playing sports as a criterion for Physical Exercise). However, because of ethical/legal concerns, we could not implement appropriate criteria for certain fundamental motives (e.g., Sex). For other fundamental motives, we considered some items that could assess appropriate criteria to be either too similar to 16mrs items with respect to their wording (e.g., “How often do you take revenge?”) or too abstract to be properly answered (e.g., “How often do you seek praise a week?”). Therefore, for the Social Acceptance, Autonomy, Structure, Revenge, and Sex scales, we could not provide criteria that could be reasonably assessed *via* an online survey. For most scales, however, we found appropriate criteria, which are displayed in Table 2.

#### Data Analysis

##### Outlier analysis

We conducted an outlier analysis by computing Mahalanobis distance as described by Tabachnick and Fidell (2014) and Kline (2015). For the *p*-value corresponding to the χ^2^ value, we used the very conservative cutoff of 0.001 to identify outliers as recommended. The procedure identified 76 cases as outliers, which were subsequently removed from the data set. Thus, *N* = 923 participants were provided for the analyses.

##### Mokken scale analysis

To cross-validate the results from Study 2, we investigated whether the *H*_*i*_ and *H* indices also exceeded the respective cutoffs in a different sample. To this end, we specified each of the 16 scales in an MHM (Mokken, 1971) for polytomous items (Hemker et al., 1997) to check if the computed scalability indices from Study 2 would hold in a different sample. To examine the assumptions of latent monotonicity and local independence, we again used the restscore method (Van der Ark, 2012) and the *W*^(1)^ index (Straat et al., 2016). To judge the resulting scales, we applied Mokken’s (1971) cutoffs by which a scale was considered weak if 0.3 ≤ *H* < 0.4, moderate if 0.4 ≤ *H* < 0.5, and strong if *H* > 0.5.

##### Reliability

We calculated reliability coefficients with LCRC (van der Ark et al., 2011) and coefficient alpha.

##### Validity

###### Convergent and discriminant validity

To compare the 16mrs with the respective LUXXprofile scales, we calculated Spearman rank correlations. Relationships between the 16 fundamental motives and the explicit UMS-6 motives as well as the BFI-2 traits were investigated by computing Spearman rank correlations. In accordance with Cohen (1988), we interpreted correlation coefficients 0.10 < *r* < 0.30 as small effects, 0.30 ≤ *r* < 0.50 as medium-sized effects, and *r* ≥ 0.50 as large effects.

###### Criterion validity

We computed regression analyses to predict the behavioral indicators using the respective 16mrs motive scores as well as age, gender, and education as predictors. Gender (0 = *female*, 1 = *male*) and education (0 = *low education*, 1 = *high education*) were entered into the regression analyses as dummy-coded predictors. Because the behavioral indicators were measured with different types of variables (i.e., dichotomous, metric, and count), we used different regression analysis techniques available in the stats package in R (R Core Team, 2016). We used Poisson or quasipoisson regression for count variables that constituted the criterion for Curiosity, Status, Retention, Social Participation, Physical Exercise, and Family. Dichotomous outcomes for Dominance, Idealism, and Safety were analyzed with binomial logistic regression. For analyses involving binomial logistic, Poisson, and quasi-Poisson regression, we provided odds ratios as a measure of the impact of the predictors. We analyzed the metric outcome for Morality with multiple linear regression.

We also computed coefficients of determination, which are appropriate for the different regression techniques. We computed adjusted *R*^2^ values for multiple linear regression, *R^2^_*N*_* (Nagelkerke, 1991) for logistic regressions, and an adjusted version of the variance function-based coefficient of determination (Zhang, 2016) for Poisson and quasi-Poisson regression. *R*^2^ is also implemented in the R package stats (R Core Team, 2016), and for *R^2^_*N*_* and its adjusted version, we used the R package rsq (Zhang, 2017). A check of the model assumptions for the different models revealed that in some cases, the assumptions of homoscedasticity or normality of the residuals were violated. To address the subsequent invalidation of confidence intervals, we computed bias-corrected and accelerated (BCa) confidence intervals as recommended by Crawley (2013).

### Results and Discussion

#### Mokken Scale Analysis

Before interpreting the *H* indices, we investigated whether the assumptions of the MHM also held in the cross-validation sample. Using the restscore method to investigate latent monotonicity, we examined the number of violations as well as the *Crit* values (Molenaar and Sijtsma, 2000) for each item. If latent monotonicity is violated, that is, if there are local decreases in an item step response function, *Crit* values can be used to assess the seriousness of the violation. Molenaar and Sijtsma (2000) stated that *Crit* < 0.40 indicates violations due to sampling error, but these do not require an intervention; 0.40 ≤ *Crit* < 0.80 indicates mild violations, which need further considerations of pros and cons to decide whether to remove or keep an item; whereas *Crit* ≥ 0.80 indicates serious violations of latent monotonicity. Our results indicated that the assumption of latent monotonicity clearly held for 47 items, and when violations were reported, the *Crit* values were below 0.40. The only exception was Item 1 from the Morality scale. For this item, one violation was reported with a corresponding *Crit* value of 0.47. Although the value was above 0.40, we still considered the item to be acceptable because, in Study 2, no violation was reported for this item at all, and the *Crit* value was still far below 0.80.

Investigation of local independence using the *W*^(1)^ index (Straat et al., 2016) revealed no violations of this assumption whatsoever. Because the assumptions of the MHM held, the *H* indices of the 16 scales could safely be interpreted. Table 3 summarizes the *H* indices for all 16 scales as well as the means, standard deviations, and *LCRC* reliability coefficients. The *H* indices ranged from 0.32 to 0.76. Following Mokken’s (1971) rules of thumb, one scale (Autonomy) could be considered weak, four scales could be considered moderate (Social Acceptance, Retention, Morality, and Sex), and 11 scales could be considered strong (Curiosity, Dominance, Status, Social Participation, Idealism, Structure, Safety, Revenge, Physical Exercise, Food Enjoyment, and Family). Summing up the results on scalability, all scales exceeded the lower bound of 0.3 of what could be considered a useful scale.

**TABLE 3:** Descriptive statistics for the 16 scales.

**Scale**	***M***	***SD***	***H***	**LCRC**	**α**
Curiosity	3.52	0.97	0.68	0.84 (4)	0.84
Social Acceptance	2.53	0.97	0.40	0.63 (3)	0.64
Dominance	2.39	1.13	0.61	0.82 (4)	0.82
Status	2.17	1.09	0.58	0.78 (3)	0.79
Retention	3.35	0.92	0.49	0.71 (3)	0.72
Autonomy	3.47	0.77	0.32	0.52 (2)	0.55
Social Participation	2.74	1.00	0.57	0.76 (3)	0.78
Morality	3.61	0.77	0.43	0.65 (3)	0.64
Idealism	3.02	1.04	0.56	0.74 (3)	0.76
Structure	2.92	1.18	0.61	0.79 (3)	0.80
Safety	3.16	0.96	0.56	0.75 (3)	0.76
Revenge	2.11	1.19	0.53	0.74 (3)	0.75
Physical Exercise	2.58	1.30	0.76	0.87 (4)	0.89
Food Enjoyment	3.26	1.06	0.64	0.81 (4)	0.82
Family	3.65	1.04	0.62	0.80 (4)	0.81
Sex^*a*^	2.38	1.06	0.48	0.68 (3)	0.68

*^*a*^*n* = 921.*

#### Reliability

The corresponding reliability coefficients ranged from LCRC = 0.52 and α = 0.55 (Autonomy) to LCRC = 0.87 and α = 0.89 (Physical Exercise). According to the generally accepted lower bound of reliability of 0.60 for the assessment of group differences (Molenaar and Sijtsma, 2000; Murphy and Davidshofer, 2005), all scales could be considered suited for this kind of research with the exception of Autonomy. This scale experienced an unexpectedly large drop in reliability, and we therefore recommend that it be used with caution in future research. Intercorrelations between the 16mrs ranged from –0.02 (Social Acceptance and Food Enjoyment) to 0.73 (Dominance and Status). In general and in line with the sensitivity theory, fundamental motives can be expected to correlate with one another because some motives can also be a means to satisfy another motive (Havercamp, 1998). For example, following rules (Morality) can be a means to avoid stressful or potentially dangerous situations, and such avoidance in turn satisfies the Safety motive. As Dominance and Status both represent aspects of the power motive (Schönbrodt and Gerstenberg, 2012), a high correlation between these two fundamental motives was expected from a theoretical perspective. However, further investigations utilizing the Fornell–Larcker criterion (Fornell and Larcker, 1981) revealed problems to empirically discriminate the two constructs. The average absolute value of the correlation coefficients was 0.19 (*SD* = 0.12), which was slightly above the mean intercorrelation of the Reiss Profile scales of 0.15, as reported by Havercamp (1998). In general, the intercorrelations of the 16mrs differed from the intercorrelations of the Reiss Profile scales (Havercamp, 1998). In addition to this, the 16mrs were constructed on the basis of conceptually revised construct definitions (Table 1), which differ from those of the Reiss Thus, considerable differences in the correlations with the Big Five traits were expected. A table displaying the intercorrelations of the 16mrs can be obtained from the Supplementary Material.

#### Convergent and Discriminant Validity

##### Big Five Inventory-2

Table 4 presents correlation coefficients for the 16 fundamental motives and the Big Five traits. As can be seen in Table 4, we found 11 significant correlation coefficients for Extraversion, representing small to medium-sized effects. As hypothesized, the largest correlation coefficients were.47 (*p* < 0.01) with Social Participation, 0.32 (*p* < 0.01) with Dominance, and.29 (*p* < 0.01) with Physical Exercise. The only significant negative correlation emerged for Safety. No significant correlations were found for Social Acceptance, Retention, Morality, Revenge, or Sex. For Agreeableness, the correlation coefficients revealed significant small to medium-sized relationships with seven fundamental motives. On a descriptive level, the strongest positive correlations we found were 0.43 (*p* < 0.01) with Idealism and 0.34 (*p* < 0.01) with Social Participation. The strongest negative correlation was –0.33 (*p* < 0.01) with Revenge. The hypothesized correlation with Social Acceptance was not significant and also smaller than expected in terms of absolute value (–0.10, *p* = 0.166). For Conscientiousness, we found significant positive correlations with eight fundamental motives. By far, the strongest correlation was, as expected, 0.65 (*p* < 0.01) with Structure (again, descriptively speaking). In addition, moderate correlations were found with Family and Physical Exercise. For Negative Emotionality, the strongest correlation coefficient, of 0.34 (*p* < 0.01), was found with Social Acceptance. In addition, we found three small correlations, two of which were negative. For Open-mindedness, only four significant positive correlations were found: the strongest correlation was, as anticipated, with Curiosity (*r* = 0.57, *p* < 0.01); a medium-sized correlation was found with Idealism; and two small correlations were found with Morality and Autonomy.

**TABLE 4:** Spearman rank correlations for the 16 fundamental motives and Big Five traits.

**Scale**	**Extraversion**	**Agreeableness**	**Conscientiousness**	**Negative emotionality**	**Open-mindedness**
Curiosity	0.22**	0.08	0.15*	−0.19*	0.57**
Social Acc.	–0.13	–0.10	–0.04	0.34**	–0.10
Dominance	0.32**	−0.20**	0.02	0.05	0.09
Status	0.25**	–0.11	0.01	0.08	0.11
Retention	–0.06	0.01	0.25**	–0.06	0.06
Autonomy	0.19*	–0.08	0.15*	–0.05	0.23**
Social Part.	0.47**	0.34**	0.05	–0.14	0.13
Morality	0.07	0.21**	0.25**	–0.07	0.27**
Idealism	0.19**	0.43**	–0.06	–0.07	0.45**
Structure	0.19**	–0.03	0.65**	–0.06	–0.03
Safety	−0.23**	0.07	0.22**	0.08	–0.13
Revenge	0.10	−0.33**	–0.06	0.24**	–0.03
Physical Exercise	0.29**	0.17*	0.34**	−0.17*	0.02
Food Enjoyment	0.23**	0.08	0.14	–0.12	0.14
Family	0.26**	0.16*	0.33**	–0.05	0.00
Sex	0.14	–0.02	–0.04	0.13	0.11

*n = 187.*

Social Acc.: Social Acceptance; Social Part.: Social Participation.

*p < 0.05; **p < 0.01.

Summing up the results, each fundamental motive except one was significantly correlated with at least one Big Five trait. The exception was Sex, which was completely unrelated to all five traits. The correlational patterns for Dominance and Status were different enough such that we view the results as providing empirical justification for a separation of these strongly related constructs. Negative Emotionality and Open-mindedness showed the fewest correlations with the 16 fundamental motives. Of note, the complete pattern of correlations differed from the correlations between the Big Five traits and the Reiss Profile scales, as reported by Olson and Weber (2004). For example, Social Participation demonstrated a medium-sized correlation with Agreeableness and a non-significant correlation with Open-mindedness, whereas the Reiss Profile scale Social Contact was significantly correlated with Open-mindedness but not with Agreeableness. Both the 16mrs and the Reiss Profile scale showed moderate to high correlations with Extraversion. As already mentioned in the description of intercorrelations of the 16mrs, the reason for the different patterns of correlations is that we carefully revisited and redeveloped the construct definitions of the 16 fundamental motives. The fact that we found mostly small and moderate correlations and only a few high correlations is in line with McAdams’ (1995) and Winter’s (2005) arguments that motives and traits refer to different levels of personality.

##### Unified Motive Scales 6

To our knowledge, no systematic investigation of the relationships between fundamental motives on the one side and the explicit Big Three motives plus Intimacy and Fear on the other side has been conducted so far. Thus, the following results, as displayed in Table 5, considerably extend the nomological network for fundamental motives. The Power motive demonstrated large correlations with Dominance (0.76, *p* < 0.01) and Status (0.64, *p* < 0.01). Medium-sized correlations were found with Curiosity and Sex. Furthermore, seven small positive correlations were found as well as a small negative correlation with Safety (–0.20, *p* < 0.01). Achievement showed the largest number of strong correlations with the fundamental motives. The strongest correlations were found for Curiosity (0.62, *p* < 0.01), Status (0.52, *p* < 0.01), and Dominance (0.50, *p* < 0.01). Furthermore, medium-sized correlations were found for Sex and Physical Exercise. Also, six small correlations were found, two of which were negative. Affiliation showed one large correlation with Social Acceptance (0.78, *p* < 0.01). Beyond that, only eight small correlations were found, two of which were negative. Intimacy yielded a large correlation with Family (0.50, *p* < 0.01) and medium-sized correlations with Social Acceptance and Sex. Beyond that, seven small positive correlations were found. For fear, we found significant correlations with only three fundamental motives. The strongest correlation was found with Social Acceptance (0.56, *p* < 0.01). Furthermore, a medium-sized correlation was found for Safety, and a small correlation was found for Status. In sum, the results showed that each fundamental motive, except for Retention and Structure, was significantly related to at least one of the Big Three motives or Intimacy or Fear. In total, the correlational pattern provides evidence that the fundamental motives cover motivational domains that are not assessed by the Big Three or the intimacy or fear motives.

**TABLE 5:** Spearman rank correlations for the 16 fundamental motives and the motives assessed with the unified motive scales 6 (UMS-6).

**Scale**	**Power**	**Achievement**	**Affiliation**	**Intimacy**	**Fear**
Curiosity	0.35**	0.62**	0.01	–0.03	–0.04
Social Acceptance	0.12	0.11	–0.11	0.21**	0.56**
Dominance	0.76**	0.50**	0.20**	0.21**	0.09
Status	0.64**	0.52**	0.20**	0.24**	0.15*
Retention	0.07	0.12	0.06	0.02	0.06
Autonomy	0.18*	0.26**	–0.05	0.07	–0.01
Social Participation	0.17*	0.14	0.78**	0.38**	–0.11
Morality	0.01	0.15	0.12	0.27**	0.01
Idealism	0.15*	0.21**	0.08	0.25**	–0.02
Structure	0.09	0.12	0.12	0.12	–0.05
Safety	−0.20**	−0.18*	−0.16*	0.15	0.32**
Revenge	0.18*	0.16*	−0.21**	–0.03	0.04
Physical Exercise	0.23**	0.32**	0.23**	0.16*	–0.02
Food Enjoyment	0.19*	0.19*	0.20**	0.27**	–0.13
Family	0.05	0.05	0.24**	0.50**	–0.08
Sex	0.30**	0.36**	0.17*	0.30**	–0.06

n = 174.

*p < 0.05; **p < 0.01.

##### LUXXprofile

The correlations between all 16mrs and the corresponding scales of the LUXXprofile reflected large effects.

#### Criterion Validity

Table 6 displays the results of the investigation of the criterion validity of 11 out of the 16 motives of the 16mrs. The results supported our hypotheses except for the results concerning the Family motive. For the remaining scales, the mean scores were valid predictors of the respective behavior, with effect sizes computed as odds ratios that ranged from 1.18 to 1.90 for the scales with a positive relationship with the criterion and 0.65 for Safety, which showed the expected negative relationship with the criterion. For the linear regression model for Morality, an effect size of β = –0.22 emerged. For the Family motive, the assumptions of homoscedasticity and normality of the residuals were violated, so we computed BCa bootstrapped confidence intervals. These BCa intervals, as opposed to the original intervals, included zero, rendering the effect non-significant. In general, however, the coefficients of determination were rather low with the exception of the model for Physical Exercise. This seems to be in line with the concept of equifinality that was introduced by Brunswik, as cited by Heckhausen and Heckhausen (2010). The concept states that different situations or actions can satisfy the same motives. Therefore, it is nearly impossible to find an action or situation that almost everybody strives for to satisfy the same motive. For Physical Exercise, however, the criterion seems to have the right level of abstraction. It is quite straightforward that people who have a strong motive for Physical Exercise will satisfy it with sports. The kind of sport (e.g., playing football, running, playing tennis, or working out) may vary considerably, but all kinds of sports are subsumed under the expression of sports, which therefore constitutes a good criterion. On the contrary, the number of restaurant visits in a month describes only a specific action. People with a high score on the Food Enjoyment motive may prefer to satisfy it by preparing food themselves or by taking a cooking course instead of going to a restaurant. For different actions related to the Food Enjoyment motive, no such subsuming concept exists the way it does for sports, and thus, researchers must accept that the coefficient of determination will be a lot smaller in this analysis. Likewise, this argument applies for the other analyses as well. Nevertheless, the analyses demonstrate that motives assessed with the 16mrs are systematically related to behaviors that provide one means, among others, for their satisfaction.

**TABLE 6:** Criterion validities for 11 scales of the 16 motives research scales (16mrs).

**Criterion**	**Predictor**	***b***	**β**	**95% CI**	**OR**	**Coef. of determination**
Wikipedia	**Curiosity**	0.41	0.38	0.12, 0.72^+^	1.50	0.08
	Age	0.00	0.00	–0.02, 0.02^+^	1.00	
	Gender	0.56	0.28	0.11, 1.03^+^	1.76	
	Education	0.24	0.11	–0.49, 0.89^+^	1.27	
Leadership	**Dominance**	0.38	0.41	0.20, 0.55	1.46	0.22
	Age	0.03	0.41	0.02, 0.05	1.03	
	Gender	1.07	0.54	0.73, 1.44	2.94	
	Education	1.27	0.55	0.84, 1.74	3.59	
Academ. degrees	**Status**	0.17	0.18	0.04, 0.29	1.18	0.09
	Age	0.01	0.14	0.00, 0.02	1.01	
	Gender	0.03	0.02	–0.22, 0.29	1.03	
	Education	4.26	1.84	2.83, 6.91	70.78	
Investments	**Retention**	0.17	0.15	0.06, 0.27	1.18	0.08
	Age	0.00	–0.03	–0.01, 0.00	1.00	
	Gender	0.26	0.13	0.09, 0.44	1.30	
	Education	0.00	0.00	–0.21, 0.20	1.00	
Friends	**Social Part.**	0.27	0.26	0.15, 0.41^+^	1.31	0.11
	Age	0.01	0.08	0.00, 0.02^+^	1.01	
	Gender	0.02	0.01	–0.23, 0.26^+^	0.97	
	Education	–0.03	–0.01	–0.34, 0.25^+^	1.02	
Delinquency	**Morality**	–0.24	–0.22	–0.41, –0.08^+^		0.11
	Age	0.01	0.18	0.00, 0.02^+^		
	Gender	0.33	0.20	0.11, 0.56^+^		
	Education	0.31	0.16	0.09, 0.51^+^		
Honorary office	**Idealism**	0.39	0.41	0.07, 0.72	1.47	0.11
	Age	0.02	0.28	0.00, 0.04	1.02	
	Gender	–0.14	–0.07	–0.77, 0.51	0.87	
	Education	0.66	0.29	–0.04, 1.38	1.94	
Freelance	**Safety**	–0.42	–0.40	–0.67, –0.18	0.65	0.16
	Age	0.04	0.54	[0.02, 0.06]	1.04	
	Gender	0.02	0.01	–0.44, 0.49	1.02	
	Education	1.76	0.76	0.98, 2.73	5.82	
Sports per week	**Physical Ex.**	0.64	0.82	0.53, 0.75^+^	1.90	0.47
	Age	0.00	–0.07	–0.01, 0.00^+^	1.00	
	Gender	0.26	0.13	–0.01, 0.53^+^	1.30	
	Education	0.01	0.06	–0.32, 0.33^+^	1.01	
Restaurant visits	**Food Enj.**	0.23	0.23	0.12,0.33^+^	1.25	0.07
	Age	0.00	0.05	0.00, 0.01^+^	1.00	
	Gender	0.23	0.12	–0.01, 0.47^+^	1.26	
	Education	–0.14	–06	–0.45, 0.14^+^	0.87	
Family visits	**Family**	0.22	0.24	–0.02, 0.41^+^	1.25	0.04
	Age	0.00	0.03	–0.01, 0.01^+^	1.00	
	Gender	0.21	0.11	–0.13, 0.55^+^	1.24	
	Education	0.26	0.11	–0.15, 0.63^+^	1.30	

*95% CI, 95% confidence interval;*

*^+^, bias-corrected and accelerated (BCa) bootstrap interval.*

*OR: odds ratio; Coef. of determination: coefficient of determination; Academ. degrees: academic degrees; Social Part.: Social Participation; Physical Ex.: Physical Exercise;*

*Food Enj.: Food Enjoyment.*

*n = 160–923.*

## General Discussion

Fundamental motives constitute a theoretically meaningful, self-contained classification of explicit motives. To make them readily available in research settings, we conducted the present studies for two main purposes. First, we wanted to develop a tool for the economic, reliable, and valid assessment of fundamental motives in research settings. To this end, we used two samples to construct and one sample to validate the 16mrs. Second, we wanted to validate the 16mrs and explore its nomological network by including the Big Five personality traits and the explicit Big Three motives (i.e., Power, Achievement, and Affiliation) in addition to Intimacy and Fear. Correlation coefficients with the Big Five traits as well as the explicit Power, Affiliation, Achievement, Intimacy, and Fear motives were reasonably high with respect to contextual proximity. The intercorrelations of the 16mrs scores support the idea that fundamental motives are largely distinct constructs (Havercamp, 1998). Finally, the results indicate the predictive validity of 10 of the 16 scales. Putting these results together, the 16mrs now facilitates the assessment of fundamental motives in research settings with limited resources, such as large-scale assessments and online surveys.

## Implications for Future Research

In sum, the pattern of correlational results, especially with the Big Five traits and the explicit Big Three motives in addition to Intimacy and Fear, yields important implications. On the one hand, the strong correlations that we expected with the Big Five personality traits and the explicit Big Three motives Power, Achievement, and Affiliation in addition to Intimacy and Fear on the basis of their conceptual proximity provide support for convergent validity. On the other hand, the general pattern of correlations consisting mostly of small and medium-sized effects suggests that the 16mrs scores cannot be completely reduced to the explicit Achievement, Power, Affiliation, Intimacy, and Fear motives. Hence, the 16mrs cover motivational aspects that are not covered by the Big Three and offer a more fine-grained perspective on explicit motives, subsequently offering the potential for a more detailed understanding of motivational processes. For instance, recent research has focused on the role of explicit motives in interpersonal relationships. Investigations in this field have predominantly used rather broad explicit motives such as agency (Hagemeyer et al., 2015) or social approach and avoidance motives (Nikitin and Freund, 2015, 2017). Now, the 16 fundamental motives can add to this research by providing a more fine-grained and comprehensive perspective. For instance, investigators may wish to examine the roles of potentially conflicting motives such as Social Participation and Revenge in peer-group processes or intimate relationships or to identify motivational profiles that facilitate or prevent the establishment of social relations. In sum, fundamental motives constitute an alternative to more established personality frameworks when personality constructs serve as an antecedent or outcome in research on interpersonal relationships but also on work, education, life satisfaction, and other important areas of life.

On a more general level, important implications for using short scales such as the 16mrs should be pointed out. The application of short scales has been extensively discussed in the literature (for a summary of the pros and cons of short scale use, see Ziegler et al., 2014). As a compromise in this discussion, on the one hand, the use of short scales is generally accepted in research settings on a group level. This is especially true for large-scale assessments (Rammstedt and Beierlein, 2014). On the other hand, short scales should not be used for decision-making on an individual level (Rammstedt and Beierlein, 2014). Hence, we encourage the use of the 16mrs in research settings, but we strictly discourage its application in individual decision-making.

Another aspect to bear in mind is the comparison between online and paper-pencil surveys. Given that the 16mrs were constructed and validated exclusively with online surveys, the presented results hold only for this research environment. Although Davidov and Depner (2011) established scalar measurement invariance between online and paper-pencil versions of the Portrait Value Questionnaire, they found latent mean differences between the two versions. Likewise, Ward et al. (2014) found manifest mean score differences that resembled small effects according to Cohen’s *d* between online and paper–pencil versions of several questionnaires. Such differences might occur because participants who take online surveys seem to feel their privacy is better protected than those who answer paper–pencil surveys, and thus, online assessments are less affected by social desirability (Richman et al., 1999; Davidov and Depner, 2011). These studies show that different results between online and paper–pencil surveys may occur on the basis of different levels of susceptibility to social desirability. We do not wish to discourage the use of paper–pencil versions of the 16mrs, but we want to raise awareness that the results might not be entirely comparable to those obtained in the presented studies.

## Limitations

The present study has several methodological strengths, including the three representative samples, an item selection procedure combining the data-driven strength of ACO, and content considerations, as well as an extensive (cross-)validation of the constructed scales. However, we also need to discuss some limitations that should be taken into consideration in future research. With respect to the reliabilities of the scale scores, the coefficients generally fell in a range that could be considered acceptable to good. In comparison with instruments developed for individual decision–making, such as the Reiss Profile (Havercamp, 1998; Reiss and Havercamp, 1998) and the LUXXprofile (Kemper et al., 2017), most of the coefficients in the current studies fell in the reliability range of these two considerably longer instruments, indicating that the shortness of the 16mrs is accompanied by only a moderate decline in reliability. Furthermore, reliability estimates of the 16mrs are similar to those of the UMS-3, which assesses the same explicit motives as the UMS-6 but with three items per scale (Schönbrodt and Gerstenberg, 2012). The reliability of the Autonomy scores, however, dropped in the cross-validation beyond the desired cutoff of 0.60. Under these circumstances, researchers should exercise caution when using the Autonomy scale. However, as seen when comparing results in Samples 2 and 3, reliabilities are subject to certain fluctuations, which might again push the reliability of Autonomy scores above 0.60 in a different sample. Therefore, from a practical point of view, if one is interested in the whole framework of the 16 fundamental motives, it might be viable to also use the Autonomy scale and check whether the reliability is high enough to interpret the results. If one is particularly interested in the Autonomy scale, using this scale might be too much of a risk, and one should consider alternative scales. Furthermore, on a more general level, the methods used to investigate reliability themselves might be worthy of discussion. Sandy et al. (2016) recommended that researchers do not rely on measures of internal consistency but that they consider other coefficients of reliability such as test–retest correlations when investigating the reliability of such short questionnaires. However, if no test–retest sample is available, researchers have no choice but to rely on measures such as the LCRC and coefficient alpha, which can be computed when only a single measurement occasion is used. Nevertheless, we recommend the investigation of test–retest correlations in future studies. With respect to predictive validity, we were able to provide results for only 11 out of the 16 scales. Although a comprehensive investigation would surely be desirable, the investigation of predictive validity is only one part among several aspects of validity, for instance, convergent and discriminant validity. In sum, we view the results as supportive of the validity of the 16mrs scores.

As we assessed both the predictors and the criteria for investigating criterion validity using self-report measures, two concerns should be discussed, namely, (1) the possibility of inflated correlations due to common method bias and (2) the fact that some criteria (e.g., Delinquency) might have been subject to socially desirable responding. When assessing the predictor and criterion with the same method (i.e., questionnaires in the present study), inflated correlations resulting from common method bias are a concern. However, there is evidence that the inflating effects due to common method bias have far less impact on same-method correlations than long believed and that same-method observed correlations provide reasonably accurate representations of true score correlations (Conway and Lance, 2010; Lance et al., 2010). Concerning social desirability, we cannot rule out the possibility that some analyses, such as involving Delinquency as a criterion for the Morality scale, might have been biased. Alongside the finding that not all behavioral indicators worked as intended (see sections “Results and Discussion” in Study 3), there is a need for additional analyses in future studies involving behavioral indicators for all motives as well as less specific behavioral indicators in order to avoid problems with equifinality (i.e., different situations or actions can satisfy the same motives). To avoid potential social desirability issues, the use of objective indicators such as salary is encouraged for future studies. Future research might also use such indicators to compare the 16mrs with other motive questionnaires in terms of predictive validity.

Concerning the intercorrelations of the motives, the analyses revealed a high correlation of the Dominance motive and the Status motive. Although this has been expected from a theoretical perspective, further analyses revealed issues to empirically discriminate the two constructs. About potential reasons for this overly strong association between the two scales can only be speculated at this point. However, as this correlation that we obtained in Study 3 is in line with the results from Study 2, a sample specificity of the results seems unlikely. Moreover, analyzing the wording of the items that we developed did not indicate a strong overlap. However, compared to the dominance scale of the dominance, prestige, and leadership questionnaire (DoPL; Suessenbach et al., 2019), the dominance scale of the 16mrs could be modified in order to better reflect the aspect of behaving aggressively in order to bend others to one’s will. A revision of the wording along these lines might help to reduce the association between the two scales. As there are still theoretical justifications for distinguishing the two motives (Suessenbach et al., 2019), we would recommend revising the wording in a future study rather than merging the two scales. Moreover, depending on the aim of the researcher and the statistical framework used to model the motives, a hierarchical approach might be utilized to model the two motives as facets of a common higher order factor. This would be in line with recent results on the facets of the Power motive (Suessenbach et al., 2019). However, further investigations are needed to provide support for such an approach. For now, the results of the two scales should be interpreted with caution.

A final but practical limitation is the fact that, so far, we have provided only an extensive validation for the German version of the instrument. Although the items have already been translated into English (see Supplementary Material), we saved an extensive validation and investigations of measurement invariance for future research. A validation of the English version to render the English items equally ready to use will allow for cross-language and cross-cultural comparisons of important extensive motivational structures beyond the Big Three. However, we recommend that these types of investigations be conducted before any results from the administration of the English language version of the instrument are interpreted. A related point is also the fact that the current results relied heavily on German samples. Therefore, investigations in other cultures are needed to examine the generalizability of the current results.

## Conclusion

In summary, we used powerful state-of-the-art scale development techniques to provide a new and extensively validated set of 16 short scales for the assessment of fundamental motives. In this, the 16mrs represent an assessment approach that is specifically tailored to research purposes. The 16mrs follow Bilsky’s (2006) call for research on more comprehensive explicit motive structures that go beyond the Big Three. He argues that focusing too much on the Big Three motives leads to bias such that explicit motives such as Curiosity, Structure, and many others are neglected. The 16mrs now provide a readily available solution to counteract this bias. It is our hope that the 16mrs will help shift the focus of motive research so that it will reach beyond the Big Three and shed light on aspects of the human motive structure that have not been intensively investigated so far.

## Data Availability Statement

The datasets presented in this study can be found in online repositories. The names of the repository/repositories and accession number(s) can be found below: The datasets analyzed for this study can be found in the Open Science Framework (OSF), https://osf.io/su5mx/?view_only=ea28191f01af495aa3e1e213678f4b43. Some of the data used in Study 1 and subsets of data used in Study 2 cannot be shared due to intellectual property reasons. The code for the Ant Colony Optimization algorithm can also not be shared, because even though we were allowed to use the syntax, we do not hold any copyright for it.

## Ethics Statement

For the two independent samples analyzed in Studies 1 and 2, the participants were sampled by the private survey institute Respondi AG (respondi.com), which is based in Germany. Respondi complies with ISO 26362 (International Organization for Standardization, 2009) and ESOMAR (esomar.org) standards to ensure that data collection, storage, and processing is carried out in a safe and ethical manner. Participants for Study 3 were sampled by the private survey institute forsa main (forsa.de), based in Germany. This independent institute conducts surveys for research, political, and state institutions as well as for companies. Forsa is also a member of ESOMAR (esomar.org). All data were fully anonymized by the private survey institutes before all authors had access to them. The participants were informed that the results of this survey would potentially be used for academic publications.

## Author Contributions

JD contributed to the conceptualization, formal analysis, and writing – original draft preparation. SG contributed to the project administration and writing writing – review and editing. CN contributed to the supervision and writing – review and editing. All authors contributed to the article and approved the submitted version.

## Conflict of Interest

The authors declare that the research was conducted in the absence of any commercial or financial relationships that could be construed as a potential conflict of interest.

## Footnotes

1 forsa.de

2 esomar.org
