# Supplementary material for: Assessing 16 Fundamental Motives With Fewer Than 50 Items: Development and Validation of the German 16 Motives Research Scales (16mrs)
Source: Front Psychol. 2021 Mar 8;12:562371. doi: 10.3389/fpsyg.2021.562371 (PMC7982535; doi:10.3389/fpsyg.2021.562371)
Supplement: Supplementary file 1 [file Table_1.docx]

*Supplementary Material for:*

**Assessing 16 Fundamental Motives With Fewer Than 50 Items: Development and Validation of the 16 Motives Research Scales (16mrs)**

Note: This supplementary material is intended to appear only on the website linked to the article, and is not intended for the printed article.

**Table of Content**

[Construct Definitions and Development of the Item Pool 2](#_Toc1058860)

[Construct Definitions 2](#_Toc1058861)

[Development of the Item Pool 5](#_Toc1058918)

[Sampling Process and additional Demographic Data 6](#_Toc1058919)

[Sampling Process 6](#_Toc1058920)

[Additional Demographic Data 7](#_Toc1058924)

[Data Analysis 9](#_Toc1058930)

[Outlier Analysis 9](#_Toc1058931)

[Exploratory Factor Analysis (EFA) 10](#_Toc1058934)

[Item Selection with Ant Colony Optimization (ACO) and Mokken Scale Analysis 11](#_Toc1058935)

[Ant Colony Optimization (ACO) 11](#_Toc1058936)

[Mokken Scale Analysis 12](#_Toc1058944)

[German and English Version of the 16mrs 13](#_Toc1058950)

[Intercorrelations of the 16mrs Scales 17](#_Toc1058951)

[References used in the Online Supplementary Material 18](#_Toc1059107)

[Footnotes 21](#_Toc1059108)

[Appendix: Literature analyzed for the Revision of the Construct Definitions 22](#_Toc1059109)

# Construct Definitions and Development of the Item Pool

## Construct Definitions

As the existing construct definitions revealed room for improvement, we wanted to take the opportunity to develop a new test to further improve the fit between the construct definitions and the items as carried out in our research. Of note, there seems to be a mismatch between the original definitions of the 16 motives and the respective items (as far as they are published, for instance, in Havercamp, 1998 and as far as this can be evaluated given that not all items are published). For example, the defintion of the Tranquility motive (i.e., Need to avert anxiety and fear) covers the avoidance of anxiety and fear (Reiss, 2004), whereas the items from the Reiss Profile also tap into the avoidance of pain and unpleasant body states (Havercamp, 1998). For the Power motive, two considerably different definitions exist. One definition (Reiss, 2004) reflects the Reiss Profile item contents well (Havercamp, 1998) because it covers the need to influence others and to lead. However, in a different publication (Reiss, 2000), aspects such as seeking challenges and excellence, which seem to belong instead to the Achievement motive, were mixed into the definition of Power. To further improve the fit between item content and construct definitions and between different definitions that refer to the same motive, we decided to revise the existing construct definitions. To this end, we began our test development by conducting an extensive literature review of the 16 fundamental motives. We analyzed a total of 61 journal articles, book sections, books, and dissertations (for a list please see Appendix) targeting either the framework of 16 fundamental motives as a whole or single motives separately (e.g., Curiosity; see Table S1). On the basis of the results of this literature review, we revised the 16 construct definitions for every fundamental motive.

On the basis of the final selection of items, we revised our preliminary construct definitions according to the content of the items and labeled the constructs according to the literature and their content. The resulting construct definitions for each fundamental motive are presented in Table S1.

Table S1

*Construct Definitions for Each Fundamental Motive and the References that were Incorporated into the Final Construct Definitions*

| Fundamental motive | Construct definition | References |
| --- | --- | --- |
| Curiosity | Interest in increasing one’s knowledge, gaining perceptions, and seeking intellectual challenges | (Murray, 1938; Havercamp, 1998; Reiss, 2004, 2008; Mussel et al., 2012; Mussel, 2013) |
| Social Acceptance | Being concerned with getting recognition from other people and being accepted by them | (Murray, 1938; Schlenker and Leary, 1982; Havercamp, 1998; Reiss, 2004, 2008) |
| Dominance | Being concerned with having an impact on other people and influencing people as well as processes | (Murray, 1938; McClelland, 1975; Havercamp, 1998; Reiss, 2004, 2008; Brandstätter, 2013) |
| Status | Being concerned with getting and maintaining a reputation and a prominent position in society | (McClelland, 1975; Havercamp, 1998; Reiss, 2004, 2008; Neel et al., 2015) |
| Retention | Being concerned with building up stocks and maintaining them | (Seuntjens et al., 2015) |
| Autonomy | Being concerned with being independent from other people’s impact and expectations | (Murray, 1938; Clark and Beck, 1991; Havercamp, 1998; Bieling et al., 2000; Hmel and Pincus, 2002; Reiss, 2004, 2008) |
| Social Participation | Seeking the company of and being interested in other people | (Murray, 1938; Havercamp, 1998; Reiss, 2004, 2008; Sokolowski and Heckhausen, 2010; Brandstätter, 2013; Neel et al., 2015) |
| Morality | Being concerned with social norms that apply to society and the need to comply with them | (Havercamp, 1998; Reiss, 2004, 2008) |
| Idealism | Being concerned with helping disadvantaged people and improving society | (Murray, 1938; Havercamp, 1998; Reiss, 2004, 2008) |
| Structure | Being concerned with organizing and structuring one’s environment in a simple and consistent manner | (Murray, 1938; Neuberg and Newsom, 1993; Havercamp, 1998; Reiss, 2004, 2008; Meiser and Machunsky, 2008) |
| Safety | Being concerned with having a peaceful and secure life | (Murray, 1938; Havercamp, 1998; Pud et al., 2004; Reiss, 2004, 2008; Bernstein et al., 2009; Mitchell et al., 2013) |
| Revenge | Being concerned with retaliating when wronged or insulted by others | (Murray, 1938; Havercamp, 1998; Reiss, 2004, 2008) |

*Table S1 (continued)*

| Physical Exercise | Being concerned with physical activity and exercise | (Havercamp, 1998; Reiss, 2004, 2008) |
| --- | --- | --- |
| Food Enjoyment | Being concerned with having pleasurable experiences while eating food. This motive goes beyond the bodily need of eating | (Havercamp, 1998; Bell and Marshall, 2003; Reiss, 2004, 2008) |
| Family | Being concerned with providing care for one’s family. The motive mainly refers to one’s family of origin but might also include one’s partner or offspring | (Havercamp, 1998; Reiss, 2004, 2008; Neel et al., 2015) |
| Sex | Being concerned with having sensual and erotic experiences as well as an active, fulfilling sex life | (Cooper et al., 1998; Havercamp, 1998; Reiss, 2004, 2008; Neel et al., 2015) |

## Development of the Item Pool

Using this first draft of the construct definitions, a group of seven personality and motivation experts, supported by several student assistants who were trained in item development, wrote seven to 11 positively keyed items per construct in the German language, resulting in 144 items for the first version of the preliminary item pool. The item stems that were used to construct these items asked “how much someone likes or values certain behaviors or states” as well as “how much someone dislikes or avoids them”; examples are “It is very important for me to…,” “I need…,” “I would be very upset…,” or “I try to avoid….” These items were rated on a 6-point Likert scale ranging from 0 (does not apply at all) to 5 (applies completely). For the final 16mrs, we aimed to represent each construct with three items because Gogol et al. (2014) showed that in research contexts, three-item scales provide a good balance between shortness on the one hand and content coverage as well as good psychometric properties (i.e., reliability and validity) on the other hand. Because the 16mrs was primarily intended for research purposes, we stuck to the concept of three items per scale**.**

**Sampling Process and additional Demographic Data**

## Sampling Process

For the two independent samples analyzed in Studies 1 and 2, the participants were sampled by the private survey institute Respondi AG (respondi.com), which is based in Cologne, Germany. Respondi complies with ISO 26362 (International Organization for Standardization, 2009) and ESOMAR (esomar.org) standards to ensure that data collection, storage, and processing is carried out in a safe and ethical manner. The data were fully anonymized by Respondi before all authors had access to them. The participants were informed that the results of this survey would potentially be published. The sample can be considered representative of the German population with respect to age, gender, and education.

The sampling process was based on the predefined quotas displayed in Table S2 were taken from a study called *Best For Planning* (Gesellschaft für integrierte Kommunikationsforschung mbH & Co. KG, 2015), for which the quotas were calculated on the basis of an area sample. To this end, on the basis of municipal data, Germany was divided into 53,000 areas that each contained at least 350 households. Nonoverlapping samples were randomly collected from these areas, resulting in a total sample size of 30,177 participants. This sample is considered representative of the German population and constitutes the basis for the computation of the quotas.

In both Studies, participants completed an online survey that included demographic questions and the items targeting the 16 fundamental motives. To have a broad representation of each of the 16 fundamental motives that can be used in the item selection process, we also administered the LUXXprofile^2^ (Kemper et al., 2017), consisting of 144 items. The survey institute offered every participant credits that could be traded for vouchers, money, or donations for charity.

## Additional Demographic Data

Table S2 displays additional demographic data^1^ for the samples analyzed in Studies 1 and 2. In Study 1, 323 participants were native German speakers. Of the nonnative German speakers, eight people reported having very good German language skills, whereas two reported having good German language skills. In Study 2, three participants were not native German speakers, out of which two reported having very good German language skills, and one reported having good German language skills.

Table S2

*Quotas Retrieved from* *Gesellschaft für integrierte Kommunikationsforschung mbH & Co. KG* (Gesellschaft für integrierte Kommunikationsforschung mbH & Co. KG, 2015) *and Demographic Data for the Two Independent Samples used in Studies 1 and 2*

|  |  | Study 1 | Study 2 |
| --- | --- | --- | --- |
|  | Quotas | *N* = 333 | *N* = 236 |
| Gender |  |  |  |
| Female | 49.8% | 47.7% | 49.2% |
| Age |  |  |  |
| 16-19 | 5.6% | 5.7% | 5.9% |
| 20-29 | 17.3% | 13.5% | 16.9% |
| 30-39 | 17.2% | 12.3% | 16.9% |
| 40-49 | 22.0% | 21.6% | 21.2% |
| 50-59 | 22.0% | 28.5% | 22.5% |
| 60-69 | 15.9% | 18.3% | 16.5% |
| Educational level |  |  |  |
| No school leaving qualification | 2.9% | 3.3% | 2.5% |
| Degree from lower track school | 31.5% | 35.4% | 30.9% |
| Degree from intermediate track school | 33.9% | 31.8% | 33.5% |
| Degree from higher track school | 31.7% | 29.4% | 33.1% |

*Note.* The educational levels reported in this table refer to the German school system, which typically distributes students to one of three different tracks (lower, intermediate, and higher tracks) for secondary education on the basis of their scholastic achievement. In this system, a degree from a higher track school qualifies a student for university entrance.

Table S3 displays additional demographic data for the samples analyzed in Study 3. This time, participants were sampled by the private survey institute forsa main (forsa.de), based in Frankfurt/Main, Germany.

Table S3

*Quotas Retrieved Gesellschaft für integrierte Kommunikationsforschung mbH & Co. KG* (Gesellschaft für integrierte Kommunikationsforschung mbH & Co. KG, 2015) *and Demographic Data for the Three Independent Samples used in Study 3*

|  |  | | Study 3 | | |  |
| --- | --- | --- | --- | --- | --- | --- |
|  | | Quotas | *N* **=** 999 | *n_1_* = 200 | *n_2_* **=** 199 | *n_3_* **=** 200 |
| Gender | |  |  |  |  |  |
| Female | | 49.8% | 48.7% | 49.0% | 48.7% | 49.0% |
| Age | |  |  |  |  |  |
| 16-19 | | 5.6% | 7.6% | 7.0% | 7.5% | 7.5% |
| 20-29 | | 17.3% | 15.8% | 16.5% | 15.6% | 16.5% |
| 30-39 | | 17.2% | 19.6% | 20.0% | 19.6% | 19.0% |
| 40-49 | | 22.0% | 18.9% | 18.5% | 19.1% | 19.0% |
| 50-59 | | 22.0% | 23.2% | 23.0% | 23.6% | 23.0% |
| 60-69 | | 15.9% | 14.8% | 15.0% | 14.6% | 15.0% |
| Educational level | |  |  |  |  |  |
| No school leaving qualification | | 2.9% | 2.1% | 3.0% | 1.5% | 2.5% |
| Degree from lower track school | | 31.5% | 23.4% | 23.5% | 21.1% | 23.0% |
| Degree from intermediate track school | | 33.9% | 34.0% | 33.5% | 34.7% | 34.5% |
| Degree from higher track school | | 31.7% | 40.4% | 40.0% | 42.7% | 40.0% |

*Note.* The educational levels reported in this table refer to the German school system, which typically distributes students to one of three different tracks (lower, intermediate, and higher tracks) for secondary education on the basis of their scholastic achievement. In this system, a degree from a higher track school qualifies a student for university entrance.

# Data Analysis

## Outlier Analysis

Prior to the main analysis in Studies 1 and 2, we conducted an outlier analysis by computing Mahalanobis distance as described by Kline (Kline, 2015) and Tabachnick and Fidell (Tabachnick and Fidell, 2014). For the *p*-value corresponding to the χ^2^ value, we used the very conservative cut-off of .001 to identify outliers as recommended. Table S4 displays the number of outliers removed based on the described procedure.

Table S4

*Number of outliers removed in Studies 1 and 2*

| Study | *N* | Outliers | *n*_provided_ |
| --- | --- | --- | --- |
| Study 1 | 333 | 18 (5.41%) | 315 |
| Study 2 | 236 | 12 (5.08%) | 224 |

## Exploratory Factor Analysis (EFA)

As a first step in Study 1 and 2 respectively, we conducted EFAs separately for each of the 16 scales to determine the number of factors for each construct. We followed MacCallum, Widaman, Zhang, and Hong’s (MacCallum et al., 1999) recommendations not to conduct EFAs on large batteries of variables, especially when expecting a large number of factors unless the sample size exceeds *N* = 500. Therefore, we decided to conduct the analyses scalewise. On the basis of factor analytic studies conducted by Reiss and Havercamp (Reiss and Havercamp, 1998) and Havercamp (Havercamp, 1998), we expected that a unidimensional structure would emerge for all motive scales. To investigate this hypothesis, we used principal axis factoring with a promax rotation and compared the results of the Kaiser rule (K1), the Minimum Average Partial (MAP) test, and Parallel Analysis (PA), available in the R-Menu v.2.0 (Courtney, 2013), to determine the number of factors. Because the K1 rule tends to overextract or overestimate the number of factors (Ruscio and Roche, 2012), we used it as a cap for the maximum number of factors. In cases in which only one factor was indicated by the K1 rule, we examined the eigenvalue of the second factor to avoid neglecting a hypothetical second factor with an eigenvalue barely below the cut-off of one. In cases when the K1 rule suggested more than one factor, we took the MAP and PA results into consideration to decide how many factors to extract. On the basis of the EFA results and following a common rule of thumb, we then excluded items with loadings < .32 (Tabachnick and Fidell, 2014) because items with such loadings show too little overlap with their underlying factor. We then used the remaining items for item selection. Table S4 displays the results for both studies.

Desirable outcome of the EFA was either unidimensionality or essential undimensionality (Stout, 1987, 1990) of the respective scales, which applies when exactly one major latent dimension can be distinguished from one or more minor dimensions when investigating the eigenvalues (EVs) of the first and the second factor. In Study 1, results supported a unidimensional structure for Curiosity, Retention, Idealism, Safety, Revenge, Physical Exercise, Food Enjoyment, Family, and Sex. For Social Participation (Δ_EV_ = 4.18), Dominance (Δ_EV_ = 5.22), and Structure (Δ_EV_= 4.37) the difference in EV suggested essential unidimensionality, while for the three subscales Morality (Δ_EV_ = 3.15), Status (Δ_EV_ = 3.41), and Autonomy (Δ_EV_= 2.08), the difference in EVs did not support essential unidimensionality.

In Study 2, for all fundamental motives except for Autonomy, EFA resulted in a clear one-factor structure. An examination of the EVs revealed a difference of Δ_EV_= 4.95 between the first and second factors, indicating essential unidimensionality.

# Item Selection with Ant Colony Optimization (ACO) and Mokken Scale Analysis

## Ant Colony Optimization (ACO)

In Studies 1 and 2, we used Ant Colony Optimization (ACO; 13) in combination with CFAs to select the best four items for each scale. ACO is a probabilistic algorithm based on the behavior of ants as they search for food. It is able to deal with a large number of items and finds the best combination with respect to certain criteria that are specified in an optimization function called the pheromone function. Items that comply better with these criteria accumulate pheromones more quickly than items that comply with the criteria to a lesser extent (for an example please see 16).

We set up a pheromone function that was capable of accomplishing two goals simultaneously. First, to ensure that selected items would constitute a good representation of the entire respective construct, the pheromone function should select items that, when combined into a scale, would be strongly correlated with an external criterion that broadly represents the respective constructs. As such, we chose the LUXXprofile (Kemper et al., 2017). As each of the LUXXprofile scales consists of nine items, it can be considered a broad representation of each fundamental motive. Second, the items should fit a CFA model (see Figure E1) by meeting the strict criteria: CFI > .95 and RMSEA < .05 (Hu and Bentler, 1999). The pheromone function combining these objectives is presented in Equation 1.

$\varphi_{Fit}=0.5\left( CFI-RMSEA \right)+ 0.5{Cor}_{LUXXprofile scale}$ (1)

Although we wanted the final scales to contain three items, we set up models with four indicators to get overidentified models, which then allowed us to compute fit indices. We selected the best three out of four items in an additional step in Study 2. To avoid a solely data-driven selection procedure, ACO solutions were investigated when all major aspects of the construct, as specified in the first draft of the construct definitions, were included. Additionally, we checked all ACO solutions for correlated errors due to substantial overlap between items.


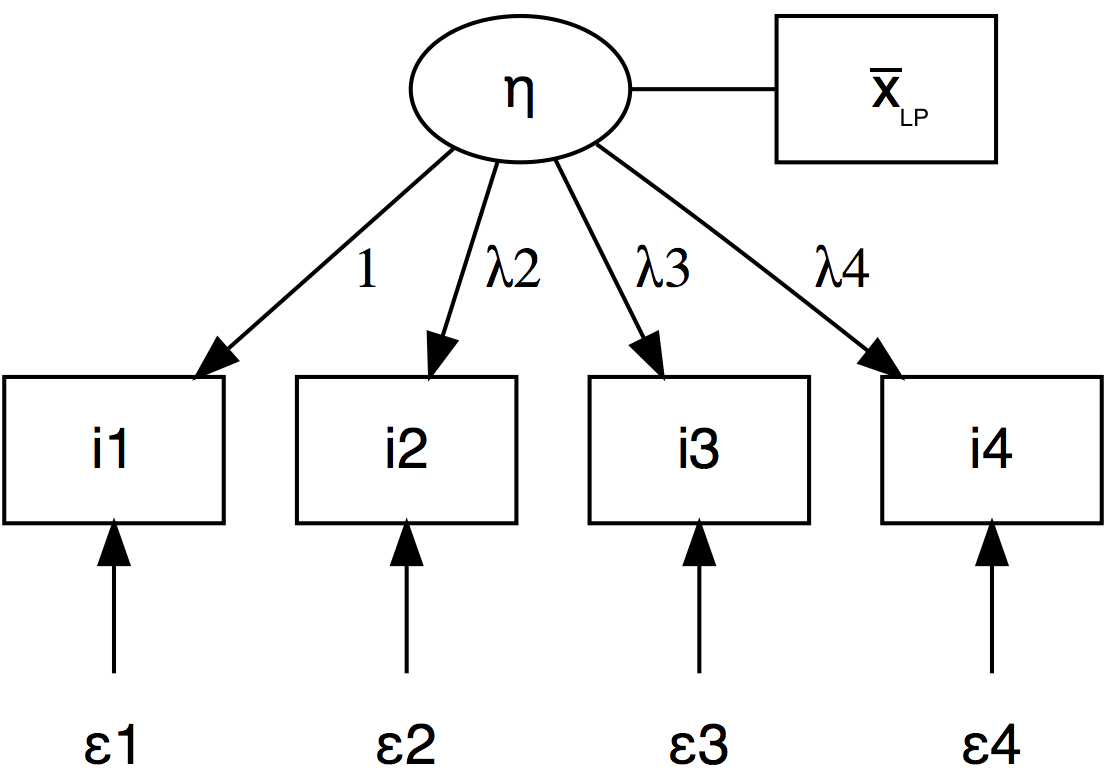


*Figure S1*. CFA model used for item selection with x̅_LP_ as the manifest

mean of the respective LUXXprofile scale.

## Mokken Scale Analysis

Following the application of ACO, we used Mokken scale analysis so that, out of each set of four items, we could select the three that could best discriminate between people with a high and a low standing on the respective motive for the final scales. In contrast to CFA, for the three-item scales, the Mokken scale analysis had no identification problems, which would have subsequently required the introduction of constraints such as fixed loadings. Analyses were conducted with the R package mokken (Van der Ark, 2012). We decided to use Mokken scale analysis because, as part of the nonparametric item response theory (NIRT) family, it is based on less restrictive assumptions than IRT models are (Sijtsma and Molenaar, 2002). Hences, it allows users to include more items on a scale while maintaining psychometrically desirable properties (Sijtsma et al., 2008).

We used the monotone homogeneity model (MHM; Mokken, 1971) for polytomous items (Hemker et al., 1997), for the final step in item selection (i.e., to select the best three out of the four items). If the model assumptions of unidimensionality, local independence, and latent monotonicity are satisfied, an ordinal scale for respondent measurement can be ascertained and the total scale score *X*_+_ represents a person’s standing on the measured motive. Under the MHM, we used scalability coefficients for the items *H_i_* as well as for the whole scale *H* for the item selection to ensure that the items of each scale would have enough in common to safely assume that they assessed the same motive. *H_i_* values represent the discriminatory power of an individual item, whereas *H* is the weighted mean of the *H_i_*s of the items included in the scale. In accordance with Sijtsma and Molenaar's (Sijtsma and Molenaar, 2002) recommendations, we excluded all items with *H_i_* < .3 because these items show only low discrimination power and are likely to violate the monotonicity assumption of the MHM. Simultaneously, we manually ensured that the items covered as much of the construct as possible (i.e., by avoiding redundancies). To judge the resulting scales, we applied Mokken's (Mokken, 1971) cutoffs by which a scale was considered weak if .3 < *H* < .4, moderate if .4 < *H* < .5, and strong if *H* > .5. As implied by this rule, scales were rejected if *H* < .3.

As a final step in item selection, we tested for whether the assumptions of MHM held for the constructed scales. To this end, we investigated unidimensionality by using EFA as described above. Local independence was investigated using index *W*^(1)^ (Straat et al., 2016). This index is suitable for detecting positive local dependence, which would be a violation of the local independence assumption. Because all items were positively worded and no recoding was necessary, we did not expect negative local dependence to occur and therefore did not check for it. The assumption of latent monotonicity was investigated by using the restscore method (van der Ark, 2011; Van der Ark, 2012).

# German and English Version of the 16mrs

Table S5 contains the German and (currently not yet validated) English version of the 16mrs. The items are rated on a 6-point Likert scale ranging from 0 (*does not apply at all* [trifft überhaupt nicht auf mich zu]) to 5 (*applies completely* [trifft voll und ganz auf mich zu]).

Table S5

*German and English Items from the 16mrs*

| Item code | German item | English item |
| --- | --- | --- |
| Family 1 | Meine Familie hat Vorrang vor allen anderen Dingen in meinem Leben. | My family takes precedence over everything else in my life. |
| Retention 1 | Ich finde es sehr wichtig, Geld anzusparen. | I think it's very important to save money. |
| Curiosity 1 | Es macht mir viel Freude, darüber nachzudenken, wie bestimmte Vorgänge zusammenhängen könnten. | I really enjoy thinking about how certain processes might be related. |
| Food Enjoyment 1 | Auch wenn ich für mich selbst Essen mache, gebe ich mir viel Mühe. | Even when I am making food only for myself, I give it my all. |
| Morality 1 | Es wäre mir sehr unangenehm, eine Abmachung zu brechen. | I would be very uncomfortable if I broke a deal. |
| Sex 1 | Ich lege großen Wert auf Sinnlichkeit und Leidenschaft. | Sensuality and passion are very important to me. |
| Social Participation 1 | Ich nutze jede Gelegenheit, um neue Leute kennenzulernen. | I take every opportunity to make new contacts. |
| Idealism 1 | Ich finde es schrecklich, wenn Menschen benachteiligt werden. | I find it terrible when people are disadvantaged. |
| Dominance 1 | Es gefällt mir, meine Umgebung zu kontrollieren. | I like to control my environment. |
| Structure 1 | Ich lege großen Wert auf Ordnung und Sauberkeit. | Order and cleanliness are very important to me. |
| Physical Exercise 1 | Ich brauche regelmäßig Bewegung, um mich im Alltag wohl zu fühlen. | Regular exercise is essential for me to feel comfortable in my everyday life. |

*Table S5 (continued)*

| Item Code | German Item | English Item |
| --- | --- | --- |
| Structure 2 | Ordnen und Strukturieren gehören zu meinen Lieblingsbeschäftigungen. | Creating order and structure are among my favorite pastimes. |

| Status 1 | Ich genieße es, wenn andere zu mir aufblicken. | I enjoy it when others look up to me. |
| --- | --- | --- |
| Idealism 2 | Es ist mir sehr wichtig, mich für andere einzusetzen. | It is very important to me to stand up for others. |
| Dominance 2 | Es gefällt mir, die Person in einer Gruppe zu sein, die entscheidet. | I love being the person in a group who makes the decisions. |
| Physical Exercise 2 | Es gefällt mir, wenn ich mich körperlich auspowern und verausgaben kann. | I enjoy being physically exhausted and expending all my energy. |
| Autonomy 1 | Ich finde es furchtbar, wenn Menschen versuchen meine Handlungen zu beeinflussen. | I think it's awful when people try to influence my actions. |
| Family 2 | Es fühlt sich großartig an, von meiner Familie gebraucht zu werden. | It feels great that my family relies on me. |
| Revenge 1 | Wenn mich jemand provoziert, muss er damit rechnen, dass ich zurückschlage. | If someone provokes me, he/she should expect me to fight back. |
| Status 2 | Ich versuche meine gesellschaftliche Stellung zu verbessern. | I try to improve my social position. |
| Revenge 2 | Ich habe manchmal Rachephantasien. | I sometimes fantasize about revenge. |
| Retention 2 | Ich gebe mir große Mühe, sparsam mit meinen Vorräten umzugehen. | I try very hard to handle my resources sparingly. |
| Morality 2 | Pflichtbewusst zu handeln ist mir äußerst wichtig. | It is very important to me to act dutifully. |
| Food Enjoyment 2 | Ich lege großen Wert auf gutes Essen. | Good food is very important to me. |
| Structure 3 | Ich lege jedes Ding immer an seinen Platz zurück. | I always put things back to where they belong. |

*Table S5 (continued)*

| Item code | German item | English item |
| --- | --- | --- |
| Autonomy 2 | Selbstbestimmt leben zu können, ist mein wichtigstes Lebensziel. | Feeling in control of my life is vital to me. |
| Social Acceptance 1 | Ich bemühe mich dauernd, keine Fehler zu machen. | I constantly try not to make any mistakes. |
| Curiosity 2 | Ich strebe nach Wissen und Erkenntnis. | I strive to acquire knowledge and make discoveries. |
| Status 3 | Ich brauche ein hohes Ansehen in der Gesellschaft, um glücklich zu sein. | I need a high standing in society to be happy. |
| Retention 3 | Ich versuche, Verschwendung zu vermeiden. | I try to avoid being wasteful. |
| Morality 3 | Mein wichtigstes Lebensziel ist, moralisch einwandfrei zu handeln. | My most important life goal is to act completely ethically. |
| Safety 1 | Ich finde es wichtig, in unbekannten Situationen immer äußerst vorsichtig zu sein. | I find it important to always be extremely careful in unfamiliar situations. |
| Social Participation 2 | Ich genieße es sehr, meine Zeit mit anderen Menschen zu verbringen. | I really enjoy spending my time with other people. |
| Safety 2 | Ich versuche ständig mögliche Risiken zu vermeiden. | I constantly try to avoid any possible risks. |
| Food Enjoyment 3 | Ich genieße meine täglichen Mahlzeiten. | I love my daily meals. |
| Sex 2 | Es würde mir sehr gefallen, sexuell anziehend zu wirken. | I would like to be sexually attractive. |
| Family 3 | Ich brauche ein harmonisches Familienleben, um glücklich zu sein. | I need a harmonious family life to be happy. |
| Dominance 3 | Ich mag es, wenn andere tun, was ich sage. | I like it when others do what I say. |
| Social Acceptance 2 | Bevor ich etwas mache, überlege ich immer, ob mich andere deswegen ablehnen könnten. | Before I do anything, I always wonder if others might refuse me. |
| Sex 3 | Ich nehme mir manchmal Zeit für die Suche nach erotischen Abenteuern. | Sometimes I take the time to seek out erotic adventures. |

*Table S5 (continued)*

| Item Code | German Item | English Item |
| --- | --- | --- |
| Social Participation 3 | Ich bin am glücklichsten, wenn ich Teil einer Gemeinschaft sein kann. | I rejoice when I can be part of a community. |
| Revenge 3 | Wenn ich merke, dass mir jemand schaden will, dann „drehe ich den Spieß um“. | If I realize that someone wants to hurt me, I "turn the tables" on the person. |
| Social Acceptance 3 | Ich finde es furchtbar, von jemandem kritisiert zu werden. | I think it's awful to be criticized by someone. |
| Autonomy 3 | Ich vermeide es, meine Entscheidungen von anderen abhängig zu machen. | I avoid basing my decisions on other people. |
| Safety 3 | Mein wichtigstes Lebensziel ist, ein ruhiges und sicheres Leben zu führen. | My most important life goal is to live a calm and safe life. |
| Curiosity 3 | Es gefällt mir sehr, mich in komplexe Sachverhalte einzuarbeiten. | I really enjoy wrapping my head around complex issues. |
| Idealism 3 | Ich denke oft darüber nach, was ich tun kann, um die Welt ein bisschen gerechter zu machen. | I often think about what I can do to make the world a little fairer. |
| Physical Exercise 3 | Körperliche Fitness ist eines meiner wichtigsten Lebensziele. | Physical fitness is one of my highest life goals. |

# Intercorrelations of the 16mrs Scales

Table S6

*Spearman Rank Correlations for the 16mrs Scales*

| Scale | 1 | 2 | 3 | 4 | 5 | 6 | 7 | 8 | 9 | 10 | 11 | 12 | 13 | 14 | 15 | 16 |
| --- | --- | --- | --- | --- | --- | --- | --- | --- | --- | --- | --- | --- | --- | --- | --- | --- |
| Curiosity (1) |  |  |  |  |  |  |  |  |  |  |  |  |  |  |  |  |
| Social Acceptance (2) | .08* |  |  |  |  |  |  |  |  |  |  |  |  |  |  |  |
| Dominance (3) | .27** | .35** |  |  |  |  |  |  |  |  |  |  |  |  |  |  |
| Status (4) | .30** | .44** | .73** |  |  |  |  |  |  |  |  |  |  |  |  |  |
| Retention (5) | .22** | .18** | .11** | .16** |  |  |  |  |  |  |  |  |  |  |  |  |
| Autonomy (6) | .36** | .15** | .18** | .15** | .25** |  |  |  |  |  |  |  |  |  |  |  |
| Social Participation (7) | .17** | .09** | .28** | .33** | .08* | .07* |  |  |  |  |  |  |  |  |  |  |
| Morality (8) | .30** | .26** | .09** | .14** | .34** | .29** | .18** |  |  |  |  |  |  |  |  |  |
| Idealism (9) | .36** | .09** | .10** | .12** | .07* | .18** | .33** | .39** |  |  |  |  |  |  |  |  |
| Structure (10) | .10** | .21** | .19** | .20** | .39** | .23** | .14** | .32** | .02 |  |  |  |  |  |  |  |
| Safety (11) | .05 | .43** | .04 | .11** | .36** | .21** | .02 | .45** | .07* | .32** |  |  |  |  |  |  |
| Revenge (12) | .12** | .23** | .34** | .30** | .09** | .27** | -.01 | .03 | -.00 | .08* | .12** |  |  |  |  |  |
| Physical Exercise (13) | .10** | .03 | .12** | .17** | .20** | .10** | .30** | .14** | .13** | .25** | .01 | -.01 |  |  |  |  |
| Food Enjoyment (14) | .20** | -.02 | .12** | .10** | .20** | .20** | .26** | .18** | .10** | .21** | .06 | -.01 | .20** |  |  |  |
| Family (15) | .09** | .19** | .14** | .14** | .16** | .19** | .30** | .37** | .21** | .27** | .32** | .08* | .13** | .23** |  |  |
| Sex (16) | .15** | .22** | .34** | .38** | .08* | .14** | .31** | .09* | .13** | .12** | .04 | .25** | .22** | .19** | .11** |  |

**p* < .05. ***p* < .01.

# References used in the Online Supplementary Material

Bell, R., and Marshall, D. W. (2003). The construct of food involvement in behavioral research: scale development and validation. *Appetite* 40, 235–244. doi:10.1016/S0195-6663(03)00009-6.

Bernstein, A., Zvolensky, M. J., Vujanovic, A. A., and Moos, R. (2009). Integrating Anxiety Sensitivity, Distress Tolerance, and Discomfort Intolerance: A Hierarchical Model of Affect Sensitivity and Tolerance. *Behav. Ther.* 40, 291–301. doi:10.1016/j.beth.2008.08.001.

Bieling, P. J., Beck, A. T., and Brown, G. K. (2000). The sociotropy–autonomy scale: Structure and implications. *Cogn. Ther. Res.* 24, 763–780. doi:10.1023/A:1005599714224.

Brandstätter, V. ed. (2013). *Motivation und Emotion: allgemeine Psychologie für Bachelor ; mit 9 Tabellen ; [Lesen, Hören, Lernen im Web]*. Berlin: Springer.

Clark, D. A., and Beck, A. T. (1991). Personality factors in dysphoria: A psychometric refinement of Beck’s Sociotropy-Autonomy Scale. *J. Psychopathol. Behav. Assess.* 13, 369–388. doi:https://doi.org/10.1007/BF00960448.

Cooper, M. L., Shapiro, C. M., and Powers, A. M. (1998). Motivations for sex and risky sexual behavior among adolescents and young adults: A functional perspective. *J. Pers. Soc. Psychol.* 75, 1528–1558. doi:10.1037//0022-3514.75.6.1528.

Courtney, M. G. R. (2013). Determining the number of factors to retain in EFA: Using the SPSS R-Menu v2. 0 to make more judicious estimations. *Pract. Assess. Res. Eval.* 18, 1–14.

Gesellschaft für integrierte Kommunikationsforschung mbH & Co. KG (2015). b4p: best for planning 2015. München: Gesellschaft für integrierte Kommunikationsfoschung mbH & Co. KG.

Gogol, K., Brunner, M., Goetz, T., Martin, R., Ugen, S., Keller, U., et al. (2014). “My Questionnaire is Too Long!” The assessments of motivational-affective constructs with three-item and single-item measures. *Contemp. Educ. Psychol.* 39, 188–205. doi:10.1016/j.cedpsych.2014.04.002.

Häder, M. (2009). *Der Datenschutz in den Sozialwissenschaften. Anmerkungen zur Praxis sozialwissenschaftlicher Erhebungen und Datenverarbeitung in Deutschland*. Berlin: Rat für Sozial- und Wirtschaftsdaten. Available at: http://www.ssrn.com/abstract=1452636 [Accessed May 5, 2018].

Havercamp, S. M. (1998). The Reiss Profile of Motivation Sensitivity: Reliability, Validity, and Social Desirability.

Hemker, B. T., Sijtsma, K., Molenaar, I. W., and Junker, B. W. (1997). Stochastic ordering using the latent trait and the sum score in polytomous IRT models. *Psychometrika* 62, 331–347. doi:10.1007/BF02294555.

Hmel, B. A., and Pincus, A. L. (2002). The meaning of autonomy: On and beyond the interpersonal circumplex. *J. Pers.* 70, 277–310. doi:10.1111/1467-6494.05006.

Hu, L., and Bentler, P. M. (1999). Cutoff criteria for fit indexes in covariance structure analysis: Conventional criteria versus new alternatives. *Struct. Equ. Model. Multidiscip. J.* 6, 1–55. doi:10.1080/10705519909540118.

International Organization for Standardization (2009). Access panels in market, opinion and social research - Vocabulary and service requirements (ISO Standard No. 26362). Available at: https://www.iso.org/standard/43521.html.

Kemper, C. J., Dörendahl, J., and Greiff, S. (2017). *Das LUXXprofile – Manual.* Esch-sur-Alzette: University of Luxembourg.

Kline, R. B. (2015). *Principles and practice of structural equation modeling*. Fourth edition. New York: The Guilford Press.

MacCallum, R. C., Widaman, K. F., Zhang, S., and Hong, S. (1999). Sample size in factor analysis. *Psychol. Methods* 4, 84.

Marcoulides, G. A., and Drezner, Z. (2003). Model Specification Searches Using Ant Colony Optimization Algorithms. *Struct. Equ. Model. Multidiscip. J.* 10, 154–164. doi:10.1207/S15328007SEM1001_8.

McClelland, D. C. (1975). *Power: the inner experience*. New York: Irvington Publishers.

Meiser, T., and Machunsky, M. (2008). The Personal Structure of Personal Need for Structure. *Eur. J. Psychol. Assess.* 24, 27–34. doi:10.1027/1015-5759.24.1.27.

Mitchell, M. A., Riccardi, C. J., Keough, M. E., Timpano, K. R., and Schmidt, N. B. (2013). Understanding the associations among anxiety sensitivity, distress tolerance, and discomfort intolerance: A comparison of three models. *J. Anxiety Disord.* 27, 147–154. doi:10.1016/j.janxdis.2012.12.003.

Mokken, R. J. (1971). *A Theory and Procedure of Scale Analysis*. Berlin: De Gruyter.

Murray, H. A. (1938). *Explorations in personality*. Oxford: Oxford University Press.

Mussel, P. (2013). Intellect: A theoretical framework for personality traits related to intellectual achievements. *J. Pers. Soc. Psychol.* 104, 885–906. doi:10.1037/a0031918.

Mussel, P., Spengler, M., Litman, J. A., and Schuler, H. (2012). Development and Validation of the German Work-Related Curiosity Scale. *Eur. J. Psychol. Assess.* 28, 109–117. doi:10.1027/1015-5759/a000098.

Neel, R., Kenrick, D. T., White, A. E., and Neuberg, S. L. (2015). Individual differences in fundamental social motives. *J. Pers. Soc. Psychol.* 110, 887–907. doi:10.1037/pspp0000068.

Neuberg, S. L., and Newsom, J. T. (1993). Personal need for structure: Individual differences in the desire for simpler structure. *J. Pers. Soc. Psychol.* 65, 113. doi:10.1037/0022-3514.65.1.113.

Olaru, G., Witthöft, M., and Wilhelm, O. (2015). Methods matter: Testing competing models for designing short-scale Big-Five assessments. *J. Res. Personal.* 59, 56–68. doi:10.1016/j.jrp.2015.09.001.

Pud, D., Eisenberg, E., Sprecher, E., Rogowski, Z., and Yarnitsky, D. (2004). The tridimensional personality theory and pain: harm avoidance and reward dependence traits correlate with pain perception in healthy volunteers. *Eur. J. Pain* 8, 31–38. doi:10.1016/S1090-3801(03)00065-X.

Reiss, S. (2000). A mindful approach to mental retardation. *J. Soc. Issues* 56, 65–80.

Reiss, S. (2004). Multifaceted Nature of Intrinsic Motivation: The Theory of 16 Basic Desires. *Rev. Gen. Psychol.* 8, 179–193. doi:10.1037/1089-2680.8.3.179.

Reiss, S. (2008). *The normal personality: a new way of thinking about people*. Reprinted. Cambridge: Cambridge Univ. Press.

Reiss, S., and Havercamp, S. M. (1998). Toward a comprehensive assessment of fundamental motivation: Factor structure of the Reiss Profiles. *Psychol. Assess.* 10, 97. doi:10.1037/1040-3590.10.2.97.

Ruscio, J., and Roche, B. (2012). Determining the number of factors to retain in an exploratory factor analysis using comparison data of known factorial structure. *Psychol. Assess.* 24, 282–292. doi:10.1037/a0025697.

Schlenker, B. R., and Leary, M. R. (1982). Social Anxiety and Self-Presentation: A Conceptualization and Model. *Psychol. Bull.* 92, 641–669. doi:10.1037/0033-2909.92.3.641.

Seuntjens, T. G., Zeelenberg, M., van de Ven, N., and Breugelmans, S. M. (2015). Dispositional greed. *J. Pers. Soc. Psychol.* 108, 917–933. doi:10.1037/pspp0000031.

Sijtsma, K., Emons, W. H. M., Bouwmeester, S., Nyklíček, I., and Roorda, L. D. (2008). Nonparametric IRT analysis of Quality-of-Life Scales and its application to the World Health Organization Quality-of-Life Scale (WHOQOL-Bref). *Qual. Life Res.* 17, 275–290. doi:10.1007/s11136-007-9281-6.

Sijtsma, K., and Molenaar, I. W. (2002). *Introduction to nonparametric item response theory*. Thousand Oaks, Calif.: SAGE.

Sokolowski, K., and Heckhausen, H. (2010). “Soziale Bindung: Anschlussmotivation und Intimitätsmotivation,” in *Motivation und Handeln* (Berlin: Springer).

Stout, W. F. (1987). A nonparametric approach for assessing latent trait unidimensionality. *Psychometrika* 52, 589–617. doi:10.1007/BF02294821.

Stout, W. F. (1990). A new item response theory modeling approach with applications to unidimensionality assessment and ability estimation. *Psychometrika* 55, 293–325.

Straat, J. H., van der Ark, L. A., and Sijtsma, K. (2016). Using Conditional Association to Identify Locally Independent Item Sets. *Methodology* 12, 117–123. doi:10.1027/1614-2241/a000115.

Tabachnick, B. G., and Fidell, L. S. (2014). *Using multivariate statistics*. 6. ed., Pearson new internat. ed. Harlow: Pearson.

van der Ark, A. (2011). Getting started with Mokken scale analysis in R. *R Package Vignette URL HttpCRAN R-Proj. Orgpackage Mokken*. Available at: https://pure.uvt.nl/portal/files/1489049/MTO_Van_der_Ark_rapport_GettingStartedWithMokken_2011.pdf [Accessed June 1, 2017].

Van der Ark, L. A. (2012). New developments in Mokken scale analysis in R. *J. Stat. Softw.* 48, 1–27.

r

# Footnotes

^1^ For participants who were 16 or 17 years of age, the ability to give consent was assumed in accordance with the ethical standards for research in Germany (Häder, 2009).

^2^ We are not able to provide further psychometric information about the LUXXprofile scales due to reasons of intellectual property.

# Appendix: Literature analyzed for the Revision of the Construct Definitions (APA 6 formatted)

Ackerman, P. L. (1996). A Theory of Adult Intellectual Development: Process, Personality, Interests, and Knowledge. *Intelligence*, *22*, 227–257. https://doi.org/ 10.1016/S0160-2896(96)90016-1

Anderson, C., Hildreth, J. A. D., & Howland, L. (2015). Is the desire for status a fundamental human motive? A review of the empirical literature. *Psychological Bulletin*, *141*(3), 574–601. https://doi.org/10.1037/a0038781

Bachmann, P. (2009). *„Wirtschaft trifft Wissenschaft“: Ein Vergleich des Reiss Profile mit dem NEO-PI-R [“Economy meets science“: A comparison of the Reiss Profile and the NEO-PI-R]* (Unpublished diploma thesis). Universität des Saarlandes, Saarbrücken.

Bath, A. S. (2002). *The relationship between person-environment congruence and fundamental goals for African American and European American, female college students* (Unpublished dissertation). The Ohio State University, Columbus.

Beierlein, C., Baumert, A., Schmitt, M., Kemper, C. J., Kovaleva, A., & Rammstedt, B. (2012). *Kurzskalen zur Messung der Ungerechtigkeitssensibilität: Die Ungerechtigkeitssensibiliät-Skalen-8 (USS-8)* [Short scales for the assessment of injustice sensitivity: The injustice sensitivity scales-8]. GESIS-Working Papers 2012|21.

Bell, R., & Marshall, D. W. (2003). The construct of food involvement in behavioral research: scale development and validation. *Appetite*, *40*(3), 235–244. https://doi.org/10.1016/S0195-6663(03)00009-6

Bernstein, A., Zvolensky, M. J., Vujanovic, A. A., & Moos, R. (2009). Integrating Anxiety Sensitivity, Distress Tolerance, and Discomfort Intolerance: A Hierarchical Model of Affect Sensitivity and Tolerance. *Behavior Therapy*, *40*(3), 291–301. https://doi.org/10.1016/j.beth.2008.08.001

Bieling, P. J., Beck, A. T., & Brown, G. K. (2000). The sociotropy–autonomy scale: Structure and implications. *Cognitive Therapy and Research*, *24*(6), 763–780.

Bilsky, W., & Schwartz, S. H. (2008). Measuring motivations: Integrating content and method. *Personality and Individual Differences*, *44*(8), 1738–1751. https://doi.org/10.1016/j.paid.2008.02.001

Bless, H., Wänke, M., Bohner, G., Fellhauer, R. F., & Schwarz, N. (1994). Need for cognition: eine Skala zur Erfassung von Engagement und Freude bei Denkaufgaben [Need for cognition: A scale for the assessment of commitment and joy in cognitive tasks]. *Zeitschrift Für Sozialpsychologie*, *25*, 147–154.

Brandstätter, V., Schüler, J., Puca, R. M., & Lozo, L. (2013). Anschlussmotivation [Affiliation Motivation]. In V. Brandstätter, J. Schüler, R. M. Puca, & L. Lozo, *Motivation und Emotion [Motivation and emotion]* (pp. 42–54). Berlin, Heidelberg: Springer Berlin Heidelberg. https://doi.org/10.1007/978-3-642-30150-6_4

Chulef, A. S., Read, S. J., & Walsh, D. A. (2001). A hierarchical taxonomy of human goals. *Motivation and Emotion*, *25*(3), 191–232. https://doi.org/10.1023/A:1012225223418

Clark, D. A., & Beck, A. T. (1991). Personality factors in dysphoria: A psychometric refinement of Beck’s Sociotropy-Autonomy Scale. *Journal of Psychopathology and Behavioral Assessment*, *13*(4), 369–388. https://doi.org/10.1007/BF00960448

Cooper, M. L., Shapiro, C. M., & Powers, A. M. (1998). Motivations for sex and risky sexual behavior among adolescents and young adults: A functional perspective. *Journal of Personality and Social Psychology*, *75*(6), 1528–1558. https://doi.org/10.1037//0022-3514.75.6.1528

Cox, S. S., Bennett, R. J., Tripp, T. M., & Aquino, K. (2012). An empirical test of forgiveness motives’ effects on employees’ health and well-being. *Journal of Occupational Health Psychology*, *17*(3), 330–340. https://doi.org/10.1037/a0028314

Edwards, A. L., Crowne, D. P., & Marlowe, D. (1965). The Approval Motive: Studies in Evaluative Dependence. *The American Journal of Psychology*, *78*(3), 514. https://doi.org/10.2307/1420595

Groene, C. (2012). *Das Reiss Profile als Instrument zur Motivationsanalyse und Persönlichkeitsentwicklung potentieller Führungskräfte im Bildungsbereich [The Reiss Profile as an instrument for the motivational analysis and personality development of potential managers in the educational sector].* (Unpublished master thesis). Christian-Albrechts-Universitä, Kiel.

Havercamp, S. M. (1998). *The Reiss Profile of Motivation Sensitivity: Reliability, Validity, and Social Desirability.* (Dissertation). The Ohio State University, Columbus, Ohio.

Havercamp, S. M., & Reiss, S. (2003). A Comprehensive Assessment of Human Strivings: Test-Retest Reliability and Validity of the Reiss Profile. *Journal of Personality Assessment*, *81*(2), 123–132. https://doi.org/10.1207/S15327752JPA8102_04

Hmel, B. A., & Pincus, A. L. (2002). The meaning of autonomy: On and beyond the interpersonal circumplex. *Journal of Personality*, *70*(3), 277–310. https://doi.org/10.1111/1467-6494.05006

Hyde, M., Wiggins, R. D., Higgs, P., & Blane, D. B. (2003). A measure of quality of life in early old age: The theory, development and properties of a needs satisfaction model (CASP-19). *Aging & Mental Health*, *7*(3), 186–194. https://doi.org/10.1080/1360786031000101157

Karmann, S. (2014). *Grundlagen und theoretische Modelle des Neuromarketings und deren Anwendbarkeit im Internet am Beispiel von Motiviertes-Wohnen.de [Background and theoretical models of neuromarketing and their applicability on the internet using the example of Motiviertes-Wohnen.de].* (Unpublished master thesis). Hochschule Niederrhein, Krefeld.

Kenrick, D. T., Griskevicius, V., Neuberg, S. L., & Schaller, M. (2010). Renovating the Pyramid of Needs: Contemporary Extensions Built Upon Ancient Foundations. *Perspectives on Psychological Science*, *5*(3), 292–314. https://doi.org/10.1177/1745691610369469

Kenrick, D. T., Neuberg, S. L., Griskevicius, V., Becker, D. V., & Schaller, M. (2010). Goal-Driven Cognition and Functional Behavior: The Fundamental-Motives Framework. *Current Directions in Psychological Science*, *19*(1), 63–67. https://doi.org/10.1177/0963721409359281

Krumm, S., Grube, A., & Hertel, G. (2013). No time for compromises: Age as a moderator of the relation between needs–supply fit and job satisfaction. *European Journal of Work and Organizational Psychology*, *22*(5), 547–562. https://doi.org/10.1080/1359432X.2012.676248

Lopper, E. (2014). *„ARPI“ – Ähnlichkeitsraum von Persönlichkeitsinventaren: Systematisierung von Persönlichkeitstests mittels Card Sorting [“ARPI“ – Similarity space of personality inventories: Systematization of personality tests using card sorting].* (Unpublished bachelor thesis). Humboldt-Universität zu Berlin, Berlin.

McClelland, D. C. (1975). *Power: the inner experience*. New York: Irvington Publishers: distributed by Halsted Press.

Meiser, T., & Machunsky, M. (2008). The Personal Structure of Personal Need for Structure. *European Journal of Psychological Assessment*, *24*(1), 27–34. https://doi.org/10.1027/1015-5759.24.1.27

Meschke, U. (2011). *To what extend can the Reiss Profile help in a business context to create a measurable improvement in employee satisfaction by in- creasing their interaction and decreasing their stress levels?* (Unpublished dissertation). Liverpool John Moores University, Liverpool.

Mitchell, M. A., Riccardi, C. J., Keough, M. E., Timpano, K. R., & Schmidt, N. B. (2013). Understanding the associations among anxiety sensitivity, distress tolerance, and discomfort intolerance: A comparison of three models. *Journal of Anxiety Disorders*, *27*(1), 147–154. https://doi.org/10.1016/j.janxdis.2012.12.003

Dalbert, C., Montada, L., & Schmitt, M. (1987). Glaube an eine gerechte Welt als Motiv: Validierungskorrelate zweier Skalen [Belief in a just world: Validation correlates of two scales]. *Psychologische Beitrage*, *29*(4), 596-615. Retrieved from http://psydok.psycharchives.de/jspui/handle/20.500.11780/743

Murray, H. A. (1938). *Explorations in personality*. Oxford: Oxford University Press.

Mussel, P. (2013). Intellect: A theoretical framework for personality traits related to intellectual achievements. *Journal of Personality and Social Psychology*, *104*(5), 885–906. https://doi.org/10.1037/a0031918

Mussel, P., Spengler, M., Litman, J. A., & Schuler, H. (2012). Development and Validation of the German Work-Related Curiosity Scale. *European Journal of Psychological Assessment*, *28*(2), 109–117. https://doi.org/10.1027/1015-5759/a000098

Neel, R., Kenrick, D. T., White, A. E., & Neuberg, S. L. (2015). Individual differences in fundamental social motives. *Journal of Personality and Social Psychology*, *110*(6), 887–907. https://doi.org/10.1037/pspp0000068

Neuberg, S. L., Kenrick, D. T., & Schaller, M. (2010). Evolutionary social psychology. In S. T. Fiske, D. Gilbert, & G. Lindzey (Eds.), *Handbook of social psychology* (5th ed.). New York: John Wiley & Sons. Retrieved from http://onlinelibrary.wiley.com/doi/10.1002/9780470561119.socpsy002021/full

Neuberg, S. L., & Newsom, J. T. (1993). Personal need for structure: Individual differences in the desire for simpler structure. *Journal of Personality and Social Psychology*, *65*(1), 113. https://doi.org/ 10.1037/0022-3514.65.1.113

Preckel, F. (2014). Assessing Need for Cognition in Early Adolescence: Validation of a German Adaption of the Cacioppo/Petty Scale. *European Journal of Psychological Assessment*, *30*(1), 65–72. https://doi.org/10.1027/1015-5759/a000170

Pud, D., Eisenberg, E., Sprecher, E., Rogowski, Z., & Yarnitsky, D. (2004). The tridimensional personality theory and pain: harm avoidance and reward dependence traits correlate with pain perception in healthy volunteers. *European Journal of Pain*, *8*(1), 31–38. https://doi.org/10.1016/S1090-3801(03)00065-X

Reiss, S. (1999). The sensitivity theory of aberrant motivation. In *Anxiety sensitivity: Theory, research, and treatment of the fear of anxiety* (pp. 35–58). Mahwah, NJ.: Erlbaum.

Reiss, S. (2000). A mindful approach to mental retardation. *Journal of Social Issues*, *56*(1), 65–80. https://doi.org/ 10.1111/0022-4537.00152

Reiss, S. (2004). Multifaceted Nature of Intrinsic Motivation: The Theory of 16 Basic Desires. *Review of General Psychology*, *8*(3), 179–193. https://doi.org/10.1037/1089-2680.8.3.179

Reiss, S. (2005). Extrinsic and intrinsic motivation at 30: Unresolved scientific issues. *The Behavior Analyst*, *28*(1), 1. https://doi.org/10.1007/BF03392100

Reiss, S. (2008). *The normal personality: a new way of thinking about people* (Reprinted). Cambridge: Cambridge Univ. Press.

Reiss, S. (2009). Six Motivational Reasons for Low School Achievement. *Child & Youth Care Forum*, *38*(4), 219–225. https://doi.org/10.1007/s10566-009-9075-9

Reiss, S. (2010). *Das Reiss Profile: die 16 Lebensmotive; welche Werte und Bedürfnisse unserem Verhalten zugrunde liegen* (2. Aufl) *[The Reiss Profile: the 16 life motives; The Values and needs underlying our behavior (2nd ed.)]*. Offenbach: GABAL.

Reiss, S. (2012). Intrinsic and Extrinsic Motivation. *Teaching of Psychology*, *39*(2), 152–156. https://doi.org/10.1177/0098628312437704

Reiss, S., & Havercamp, S. M. (1996). The sensitivity theory of motivation: Implications for psychopathology. *Behaviour Research and Therapy*, *34*(8), 621–632. https://doi.org/ 10.1016/0005-7967(96)00041-1

Reiss, S., & Havercamp, S. M. (1998). Toward a comprehensive assessment of fundamental motivation: Factor structure of the Reiss Profiles. *Psychological Assessment*, *10*(2), 97. https://doi.org/10.1037/1040-3590.10.2.97

Ruch, W., Proyer, R. T., Harzer, C., Park, N., Peterson, C., & Seligman, M. E. P. (2010). Values in Action Inventory of Strengths (VIA-IS): Adaptation and Validation of the German Version and the Development of a Peer-Rating Form. *Journal of Individual Differences*, *31*(3), 138–149. https://doi.org/10.1027/1614-0001/a000022

Schlenker, B. R., & Leary, M. R. (1982). Social Anxiety and Self-Presentation: A Conceptualization and Model. *Psychological Bulletin*, *92*(3), 641–669. https://doi.org/ 10.1037//0033-2909.92.3.641

Schmidt, N. B., Richey, J. A., & Fitzpatrick, K. K. (2006). Discomfort intolerance: Development of a construct and measure relevant to panic disorder. *Journal of Anxiety Disorders*, *20*(3), 263–280. https://doi.org/10.1016/j.janxdis.2005.02.002

Schmitt, M., Baumert, A., Fetchenhauer, D., Gollwitzer, M., Rothmund, T., & Schlösser, T. (2009). Sensibilität für Ungerechtigkeit [Sensitivity for justice]. *Psychologische Rundschau*, *60*(1), 8–22. https://doi.org/10.1026/0033-3042.60.1.8

Schmitt, M., Baumert, A., Gollwitzer, M., & Maes, J. (2010). The Justice Sensitivity Inventory: Factorial Validity, Location in the Personality Facet Space, Demographic Pattern, and Normative Data. *Social Justice Research*, *23*(2–3), 211–238. https://doi.org/10.1007/s11211-010-0115-2

Seuntjens, T. G., Zeelenberg, M., van de Ven, N., & Breugelmans, S. M. (2015). Dispositional greed. *Journal of Personality and Social Psychology*, *108*(6), 917–933. https://doi.org/10.1037/pspp0000031

Simons, J. S., & Gaher, R. M. (2005). The Distress Tolerance Scale: Development and Validation of a Self-Report Measure. *Motivation and Emotion*, *29*(2), 83–102. https://doi.org/10.1007/s11031-005-7955-3

Sokolowski, K., & Heckhausen, H. (2010). Soziale Bindung: Anschlussmotivation und Intimitätsmotivation [Affiliation motivation and intimacy motivation]. In J. Heckhausen & H. Heckhausen, *Motivation und Handeln* (4. Aufl.) [*Motivation and action (4th ed.)*]. Berlin: Springer.

Stuckless, N., & Goranson, R. (1992). The Vengeance Scale: Development of a Measure of Attitudes Toward Revenge. *Journal of Social Behavior and Personality*, *7*(1).

Stumpf, H., Angleitner, A., Wieck, T., Jackson, D. N., & Beloch-Till, H. (1985). *Deutsche Personality Research Form (PRF): Handanweisung.* Göttingen: Hogrefe.

Toursel, L. (2014). *Verbesserungspotentiale im klassischen Projektmanagement über die systematische Förderung individueller und teamspezifischer Höchstleistungen mithilfe der 16 Lebensmotive nach Steven Reiss [Potential for improvement in the classical project management by the systematic promotion of individual and team specific highest performance using the 16 life motives sensu Steven Reiss].* (Unpublished bachelor thesis). Hochschule Darmstadt, Darmstadt.

Weems, C. F., Reiss, S., Dunson, K. L., Graham, R. A., Russell, J. D., Banks, D. M., & Neill, E. L. (2015). Comprehensive assessment of children’s psychological needs: Development of the child Reiss Motivation Profile for ages four to eleven. *Learning and Individual Differences*, *39*, 132–140. https://doi.org/10.1016/j.lindif.2015.03.021
